# Pathogenic *Chlamydia* Lack a Classical Sacculus but Synthesize a Narrow, Mid-cell Peptidoglycan Ring, Regulated by MreB, for Cell Division

**DOI:** 10.1371/journal.ppat.1005590

**Published:** 2016-05-04

**Authors:** George Liechti, Erkin Kuru, Mathanraj Packiam, Yen-Pang Hsu, Srinivas Tekkam, Edward Hall, Jonathan T. Rittichier, Michael VanNieuwenhze, Yves V. Brun, Anthony T. Maurelli

**Affiliations:** 1 Department of Microbiology and Immunology, F. Edward Hébert School of Medicine, Uniformed Services University of the Health Sciences, Bethesda, Maryland, United States of America; 2 Department of Chemistry, Indiana University, Bloomington, Indiana, United States of America; 3 Department of Biology, Indiana University, Bloomington, Indiana, United States of America; University of Massachusetts Medical School, UNITED STATES

## Abstract

The peptidoglycan (PG) cell wall is a peptide cross-linked glycan polymer essential for bacterial division and maintenance of cell shape and hydrostatic pressure. Bacteria in the Chlamydiales were long thought to lack PG until recent advances in PG labeling technologies revealed the presence of this critical cell wall component in *Chlamydia trachomatis*. In this study, we utilize bio-orthogonal D-amino acid dipeptide probes combined with super-resolution microscopy to demonstrate that four pathogenic Chlamydiae species each possess a ≤ 140 nm wide PG ring limited to the division plane during the replicative phase of their developmental cycles. Assembly of this PG ring is rapid, processive, and linked to the bacterial actin-like protein, MreB. Both MreB polymerization and PG biosynthesis occur only in the intracellular form of pathogenic *Chlamydia* and are required for cell enlargement, division, and transition between the microbe’s developmental forms. Our kinetic, molecular, and biochemical analyses suggest that the development of this limited, transient, PG ring structure is the result of pathoadaptation by *Chlamydia* to an intracellular niche within its vertebrate host.

## Author Summary

Pathogenic *Chlamydia* do not assemble their peptidoglycan (PG) cell wall in a classical, mesh-like sacculus, but instead apparently confine it to the mid-cell in the actively dividing, non-infectious form. We characterize the assembly and aging of this PG-ring and link its synthesis to MreB, an actin-like protein associated with lateral cell wall synthesis in bacteria. As PG is recognized by the host innate immune system, we hypothesize that the limited amount of PG synthesized by *Chlamydia* is an adaptation to the microbe’s intracellular lifestyle.

## Introduction

*Chlamydia* is an obligate intracellular pathogen and the single most prominent cause of bacterial sexually transmitted infections and infectious blindness worldwide. Frequently referred to as the ‘silent epidemic’, chlamydial infections are often asymptomatic, which results in a lengthy delay between infection and the onset of disease symptoms[1]. Approximately 1.4 million *Chlamydia* infections are reported in the United States annually[2, 3] and an estimated 90 million individuals are believed to be infected globally[4]. Untreated chlamydial genital infections can result in cervicitis, pelvic inflammatory disease, and ectopic pregnancy in women and urethritis in men.

*Chlamydia* has undergone a lengthy (>700 million year) adaptation to an intracellular environment in addition to its more recent co-evolution with humans and other vertebrate hosts [5]. As a result, pathogenic chlamydial species possess significantly smaller genomes compared to those of extracellular pathogens, free-living microbes, or environmental chlamydiae[5, 6]. *Chlamydia* exhibit a distinctive, biphasic life cycle wherein they alternate between an infectious but non-replicative elementary body (EB) and a non-infectious but replicative reticulate body (RB). Under certain conditions *Chlamydia* can differentiate into an aberrant, metabolically active but non-replicative form. These ‘aberrant bodies’ form when RBs are exposed to stressors, such as nutrient deprivation and certain antibiotics that inhibit peptidoglycan (PG) cell wall biosynthesis. Aberrant bodies exhibit a state akin to metabolic stasis that can last for days, enhancing persistence of the microbe in both human and animal hosts. When the stress is released, aberrant bodies differentiate back to RBs and normal bacterial replication continues.

**Fig 1:**
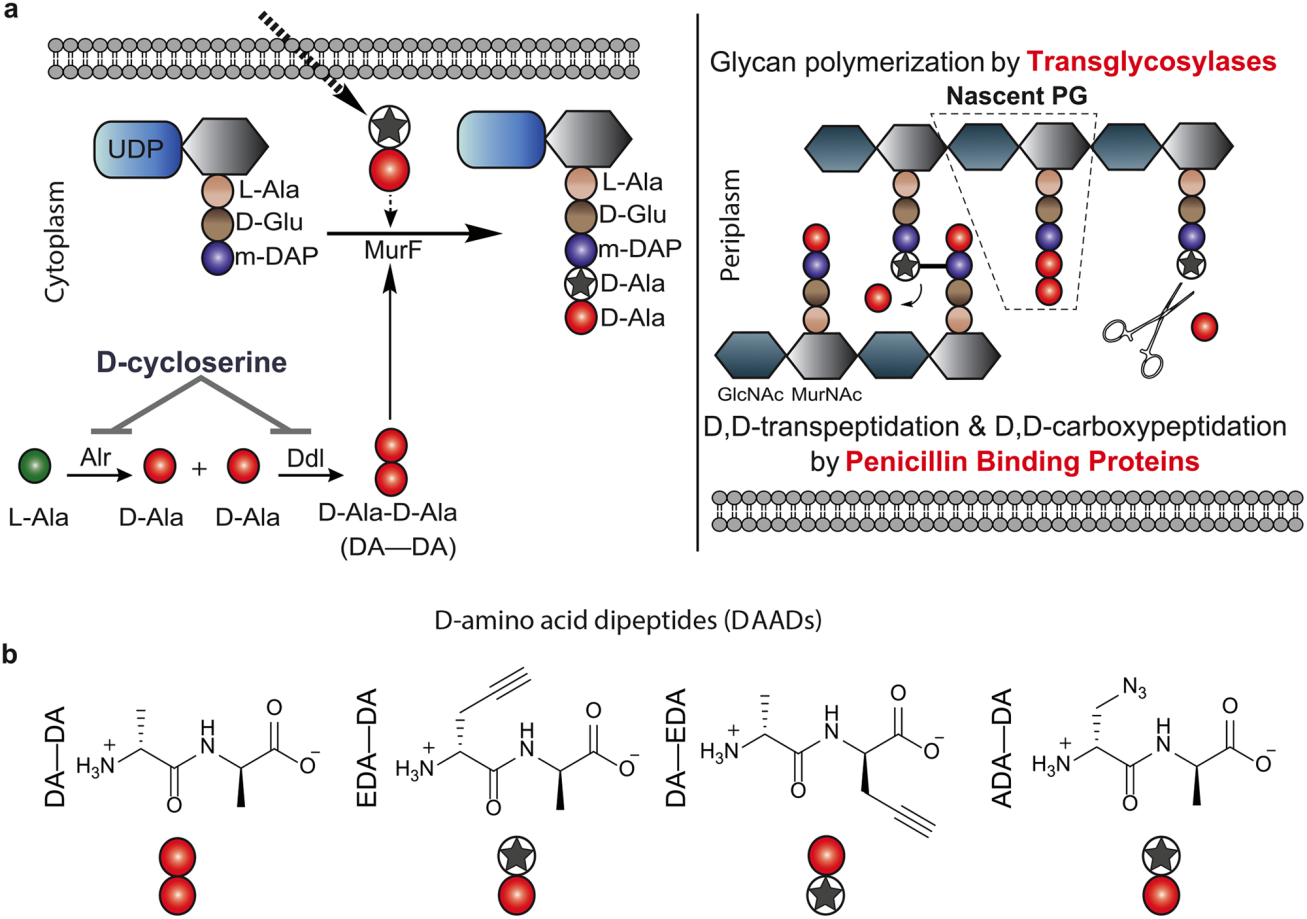
Relevant steps of PG biosynthesis and D-amino acid dipeptide (DAAD) probes used in this study. (**a**) DAADs are taken up by bacteria where they compete with endogenous D-Ala-D-Ala (DA—DA) for incorporation into PG. De novo synthesis of DA—DA is inhibited by D-cyloserine. The pentapeptide PG subunit is then flipped across the inner membrane into the periplasm where it is transglycosylated to form glycan polymers (nascent PG) and crosslinked by penicillin binding proteins (PBPs). Transpeptidation causes cleavage of the terminal D-Ala at position 5. Because the N-terminally labeled portion of DAAD becomes the amino acid at position 4 of the pentapeptide, the label is resistant to this processing and remains on the stem peptide. (**b**) PG-labeling reagents used in this study. Clickable DAADs EDA—DA and ADA—DA. Star represents the clickable amino acid.

PG is a critical cell wall component of nearly all bacteria. It is comprised of a β-(1,4) linked N-acetylglucosamine (GlcNAc) and N-acetylmuramic acid (MurNAc) disaccharide backbone and a pentapeptide stem, i.e. a muropeptide. In Gram negative and some Gram positive bacteria, the peptide stem consists of L-alanine, D-glutamate, meso-diaminopimelic acid, and a dipeptide of D-alanine-D-alanine (DA—DA) (Fig 1a). Once synthesis of the major structural component of PG (lipid II) is completed in the bacterial cytoplasm, it is flipped into the periplasm where PG assembly proceeds. Sugar moieties of the PG are initially polymerized, resulting in assembly of the nascent PG strand (Fig 1a). This step is quickly followed by cross-linking of the stem peptides from multiple strands into a structure that in the vast majority of bacteria covers the entire bacterium as a mesh-like sacculus. PG is required for cell growth and division and provides the bacterium a defined, structurally rigid and species-specific shape [7]. The unique composition of PG makes it an excellent marker for detection of bacteria by the human immune system. Indeed, PG is one of the major pathogen-associated molecular patterns (PAMPs) recognized by innate immune receptors [8].

Despite the long recognized susceptibility of pathogenic chlamydial species to common anti-PG agents such as penicillin and D-cycloserine (DCS), until recently, *Chlamydia* was thought to lack PG[9, 10]. James Moulder summed up the seemingly conflicting physiological [11–13] and biochemical [14–21] findings as the ‘chlamydial anomaly’ [22]. This paradox deepened further after the genome of *Chlamydia trachomatis* was sequenced and found to possess almost all of the genes of the PG biosynthesis pathway [23]. Numerous studies have since shown that the vast majority of the proteins encoded by these genes are functional *in vitro* or when expressed in *E*. *coli*[24–29]. The chlamydial genome appears to lack only a few PG synthesis genes, such as glutamate / alanine racemases and transglycosylases, which are essential for making PG subunits and polymerizing these subunits into PG chains, respectively [23] (Fig 1a). In addition the *Chlamydia* genome lacks a gene encoding FtsZ, a cytoskeletal cell division initiation protein that organizes numerous PG biosynthetic enzymes around the cell division plane in almost all bacteria[7].

Recently, a method was developed to label bacterial PG using the inherent promiscuity of PG biosynthetic enzymes for tagged, fluorescent D-amino acid (FDAA) probes as substrate analogs[30, 31]. Our work with the next generation of bioorthogonally tagged D-amino acid dipeptide (DAAD) probes that mimic DA—DA during PG synthesis (Fig 1a and 1b) revealed PG in *C*. *trachomatis*[9], providing the first direct evidence of its existence in these organisms. Another study confirmed the presence of PG in an environmental strain of *Parachlamydiaceae*, the amoebae-symbiont, *Protochlamydia amoebophila*[6, 10], providing evidence that both related phyla synthesize PG. Strikingly, while PG in *P*. *amoebophila* forms a typical, cell-encompassing sacculus, PG in *C*. *trachomatis* forms only a distinct ring-like band at its mid-cell.

Despite these advances, many questions remain concerning the function of PG in *Chlamydiae* and the significance of its ring-like structure. Through the use of 3D super resolution structured illumination microscopy (SIM) and clickable DAADs we define the ring-like PG structure of *C*. *trachomatis* as a ≤ 140 nm wide, dynamic ring that forms immediately after the previous cell division and follows cell constriction at the division septum. We show that this limited PG ring is also present in other pathogenic chlamydial species; *C*. *muridarum*, *C*. *caviae*, and *C*. *psittaci*. Formation of the ring is non-uniform and directly linked to the bacterial actin-like cytoskeletal protein MreB. When MreB polymerization is inhibited the PG ring is rapidly and non-uniformly turned over, suggesting competition between two coordinated, but separable processes: MreB-linked PG synthesis and an unknown turnover mechanism. We propose a reshaping model to explain how this narrow PG ring facilitates both cell enlargement and division. We also propose that *Chlamydia* limits the timing of PG ring assembly and dissociation to the intracellular replicative phase, allowing the pathogen to moderate its detection by the host immune system. These results suggest that the absence of a PG sacculus by pathogenic *Chlamydia* is the result of pathoadaptation to its intracellular niche within vertebrate hosts.

## Results

### Pathogenic *Chlamydia* lack a classical PG sacculus

**Fig 2:**
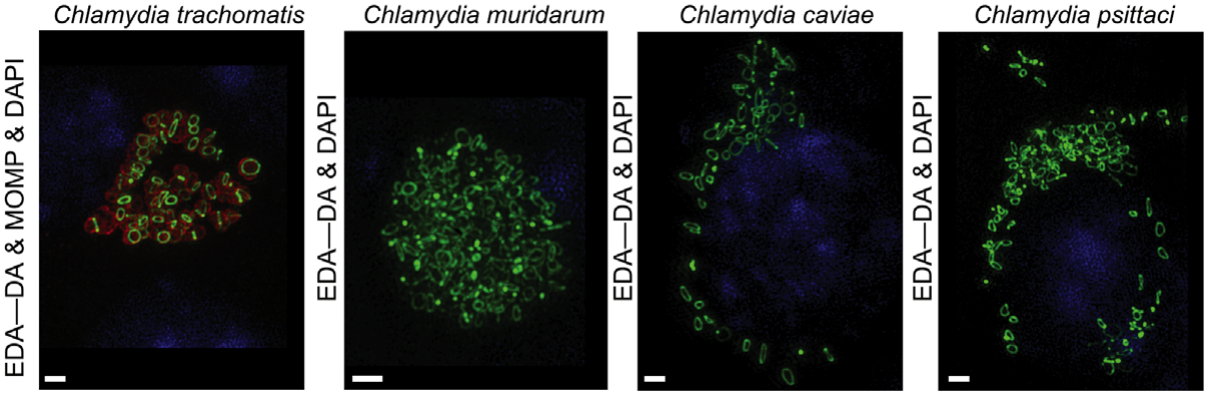
Structured illumination microscopy of DAAD PG labeling in pathogenic *Chlamydia*. Structured illumination microscopy (SIM) was conducted on four pathogenic *Chlamydia* species. EDA-DA was added 2 hours post infection (hpi) and coverslips were fixed at 18 hpi. PG labeling (represented by EDA—DA) is shown in green, the major *C*. *trachomatis* outer membrane protein (MOMP) is shown in red, and cell nuclei are in blue (this labeling scheme is maintained in all subsequent figures, unless otherwise stated). MOMP staining is not shown for the three other species. Images are representative of ~20 inclusions viewed per strain. Scale bar = 1 μm.

Despite the demonstration of a conventional PG sacculus in *P*. *amoebophila*[10], similar PG isolation and labeling techniques (e.g. using FDAAs) proved unsuccessful in studies of pathogenic *Chlamydia*[9, 16, 19]. We hypothesized that distinct morphological differences in PG configuration and structure might exist between pathogenic *Chlamydia* and the distantly related environmental endosymbionts. In our previous study we used DAADs (Fig 1b) with diffraction-limited conventional fluorescence microscopy to show that labeling of PG is constrained to a thin band in *C*. *trachomatis*[9]. This is in stark contrast to the uniform labeling of cell periphery observed in all other PG-containing bacteria that produce peripheral PG sacculi[9]. 3D super-resolution microscopy confirmed that PG from *C*. *trachomatis* grown in the presence of DAADs for several generations formed as a narrow ring (Fig 2).

This unique PG localization is common to the *Chlamydia* genus, as the DAAD-labeled PG of three evolutionarily representative, pathogenic, veterinary chlamydial species, *C*. *muridarum*, *C*. *caviae*, and *C*. *psittaci*, also localized to a single, ring-like structure (Fig 2) and none of these species had peripheral PG labeling. In contrast, DAAD labeling of the coccus-shaped *Staphylococcus aureus*, which synthesizes PG predominantly at the septum to produce a sacculus[30], resulted in uniform cell surface labeling (S1 Fig). Thus, we conclude that the restriction of DAAD labeling to a narrow ring at mid-cell is a unique and defining characteristic of pathogenic chlamydial species.

### Non-replicative chlamydial EBs do not retain PG

Evasion of the immune response is a powerful evolutionary driver for many bacterial pathogens. Because the human innate immune system recognizes and responds to PG [8], we hypothesized that Chlamydiaceae may limit PG synthesis to where and when it is absolutely needed, i.e. the division site of actively replicating cells. Since the immune system is most likely to interact with the infectious, extracellular EBs, we reasoned that *Chlamydia* may exclude PG from EBs and restrict its synthesis to the metabolically active intracellular RBs, as has been suggested previously[19, 21, 32]. This hypothesis is bolstered by the fact that the labeling of chlamydial PG was previously shown to occur no earlier than 8 hpi, coinciding with the EB to RB transition of *C*. *trachomatis* [9].

**Fig 3:**
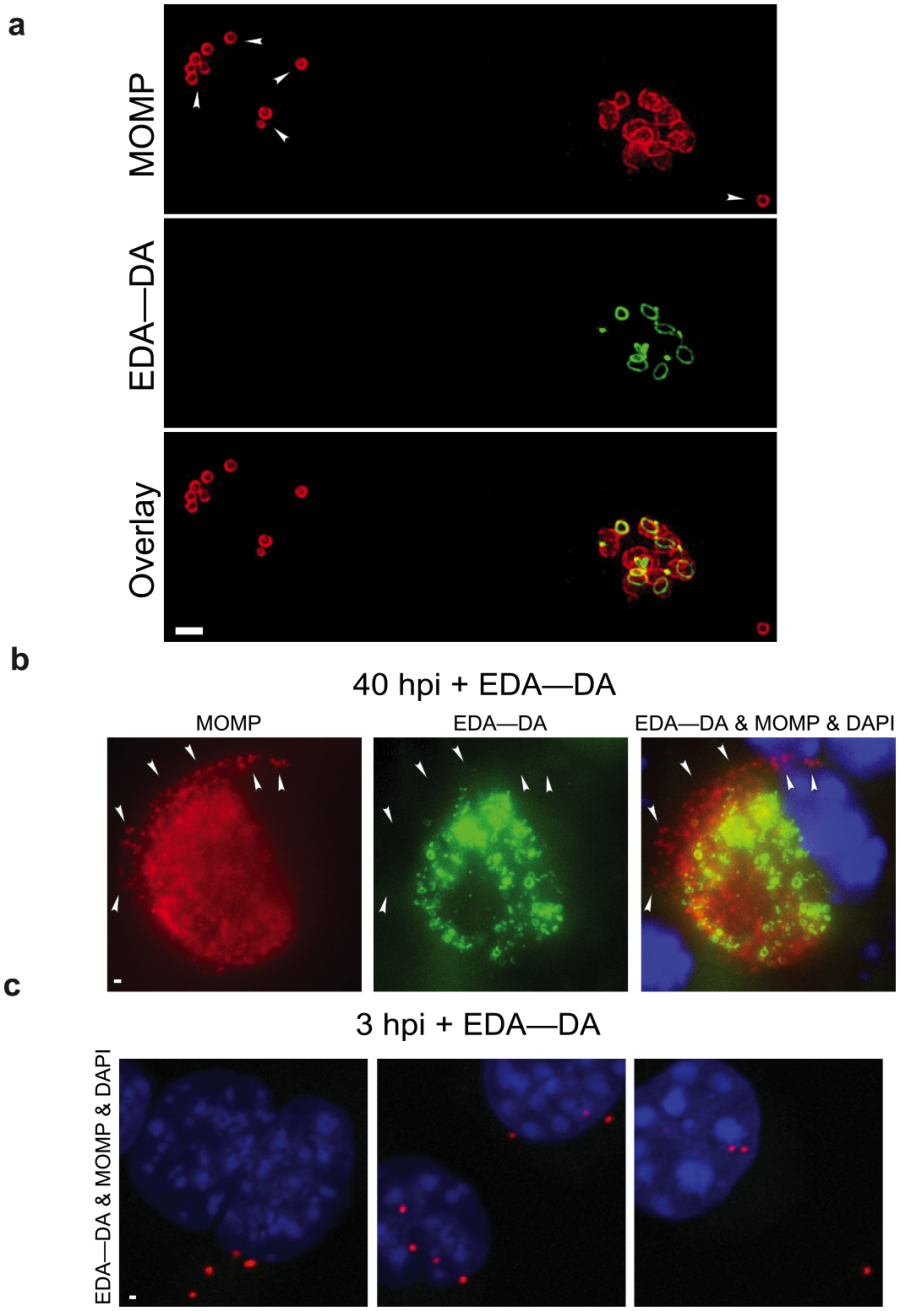
Chlamydial EBs do not retain PG labeling. **(a)** SIM of EDA—DA labeled *C*. *trachomatis* inclusions at 22 hpi. Arrowheads indicate locations of EBs. Tissue culture cells were infected with *C*. *trachomatis* and placed on a rocker for 2 hours, ensuring asynchronous infection of the cell monolayer. Bacteria were then incubated with 4 mM EDA-DA for 22 hours. **(b)** Confocal maximum intensity projections of a mature, *Chlamydia* inclusion (40 hpi) in which bacteria were grown in the presence of 4 mM EDA—DA for the entire developmental cycle. The arrowheads point to EBs (distinguished by their MOMP labeling and smaller size). **(c)** Confocal maximum intensity projections of *Chlamydia*-infected cells 4 hpi. EBs used for infection were harvested from cells that had been incubated with EDA—DA for 18 hours. Exogenous EDA—DA (4 mM) was present throughout the EB harvest as well as the subsequent reinfection. MOMP and PG labeling is the same as in Fig 2. Images are all representative of at least three separate experiments. Scale bar = 1 μm.

The use of asynchronous infections allow for the visualization of *Chlamydia* at various stages of the developmental cycle simultaneously, and the diameters of MOMP-labeled cells can be used to distinguish between RBs (≥ 1.0 μm diameter) and EBs (~0.3 μm diameter). When asynchronous infections are carried out in the presence of PG-labeling probe EDA-DA, PG labeling localizes to chlamydial RBs and appears absent in the much smaller, non-replicative EBs (Fig 3a). Given that the EBs present at 22 hpi may be noninfectious and thereby not accurately reflect the labeling potential in normal, healthy EBs, we conducted additional labeling experiments for extended durations (40 hrs), allowing enough time for completion of the *Chlamydia* developmental cycle. By 40 hpi, the vast majority of RBs containing labeled PG within mature inclusions have differentiated back into infectious EBs. In mature inclusions there was no detectable PG label in these smaller, MOMP-labeled particles (Fig 3b). We also made use of a simple, biological separation method for establishing whether *Chlamydia* EBs retained labeled PG after transitioning from replicating RBs. We infected cells with *C*. *trachomatis* for 18 hours and then incubated them in the presence of a DAAD (4 mM) for an additional 25 hours. This method allowed labeling of the bacteria in the presence of the DAAD for an entire developmental cycle, i.e. RB to EB. *Chlamydia* EBs were then harvested from mature inclusions 43 hours post infection (hpi) and used to infect a fresh cell monolayer. DAAD (4 mM) was present during all steps of collection and infection. Infected cells were then fixed at 3 hpi to ensure that only EBs were observable (RBs are incapable of invading host cells[16]) and that invading EBs did not have sufficient time to differentiate into RBs (EB to RB transition for *C*. *trachomatis* L2 strain BU/434 occurs ~8 hpi). None of the newly invading chlamydial EBs (identified by MOMP labeling) contained labeled PG (Fig 3c). These results suggest that EBs do not synthesize PG nor do they retain any PG synthesized during the RB stage.

### Replicating RBs maintain a narrow ring of PG that aligns with the septal division plane

**Fig 4:**
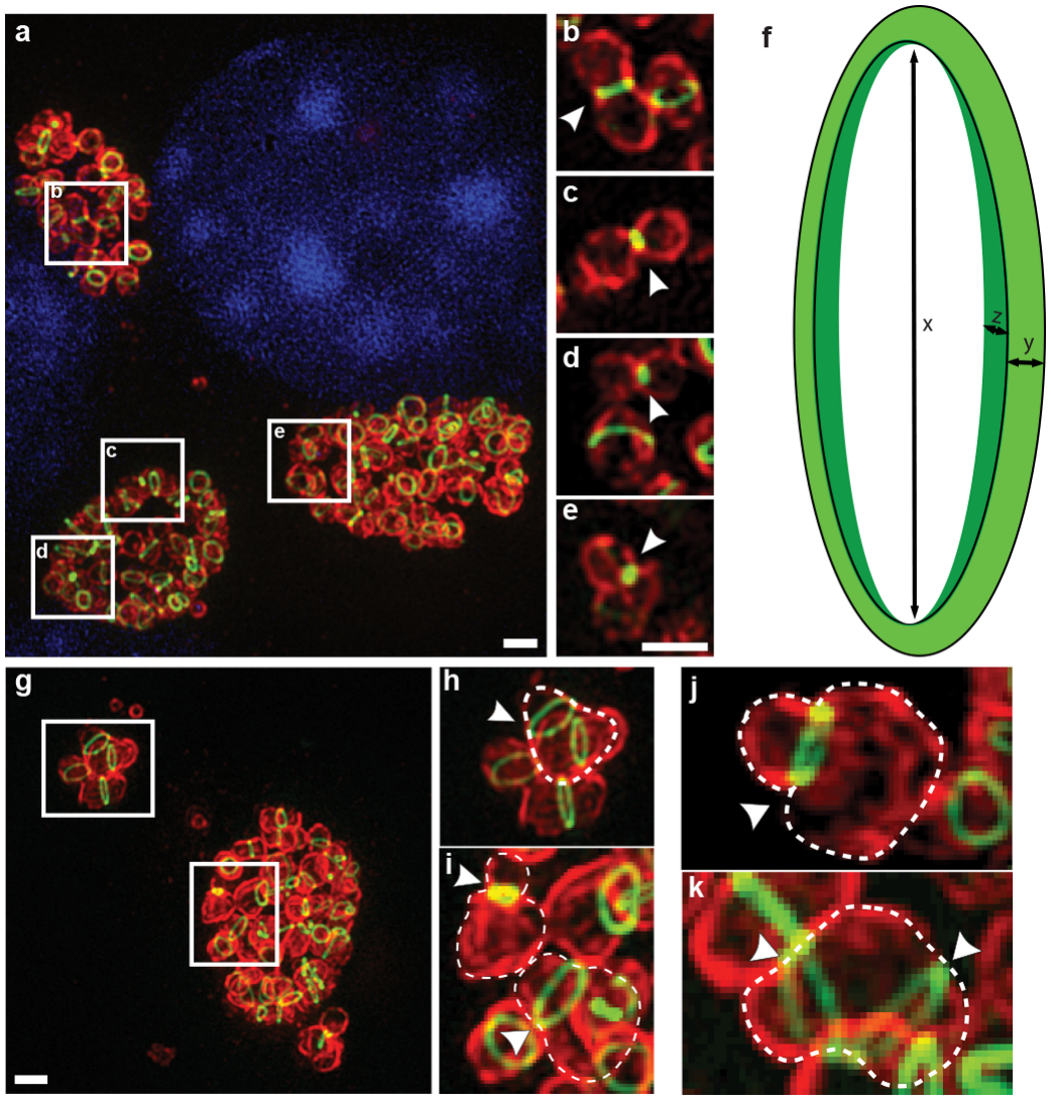
3D SIM visualization of labeled chlamydial PG. **(a-e)** Maximum intensity projection (7 μm thick Z stack) of *C*. *trachomatis* incubated with 4 mM EDA-DA at 2 hpi and fixed at 18 hpi. Panels (**b-e)** are maximum intensity projections of 0.5 μm thick planes of interest selected from panel **a**. Arrowheads indicate areas of punctate PG staining (as opposed to PG rings). **(f)** The PG ring dimensions: diameter x, width y, and thickness z. **(g-k)** 3D-SIM of cases of asymmetric cell division are outlined and marked with arrowheads for clarity. Panels **(h)** and **(i)** are magnifications of the image in panel **(g).** Images in panels **(j)** and **(k)** are independent cases. MOMP and PG labeling is the same as in Fig 2. Image is representative of ~ 20 inclusions analyzed. Scale bar = 1 μm.

The use of confocal microscopy in our previous study[9] severely limited resolution of the PG rings. In order to better characterize the localization of PG rings within chlamydial RBs, we utilized SIM microscopy to examine fluorescently labeled PG in intracellular RBs labeled for MOMP. We found that due to the compact nature of RBs within chlamydial inclusions, defining individual cell boundaries in 3D SIM projections was often challenging (Fig 4a). We decided to augment our visual characterization by also examining individual imaging planes (as opposed to rendered 3D projections). By eliminating foreground and background fluorescence contamination (Fig 4b–4e, S2 Fig, S1 Video) we were able to generate additional images that clearly delineate individual chlamydial RBs, and thereby more accurately report localization of their PG (S3 Fig, S2, S3 and S4 Videos).

Detailed, high-resolution SIM analysis of DAAD-labeled PG in *C*. *trachomatis* at 18 hpi, when the majority of *Chlamydia* within inclusions are in the actively growing/dividing RB phase, revealed the following features: 1) PG rings were confined to mid-cell: in dividing bacteria in which a membrane invagination was clearly visible, PG localized to the division septum (Fig 4a and 4b and S3a–S3d Fig); 2) PG ring diameter (x, in Fig 4f) of individual cells within a single inclusion was variable but often correlated with the diameter of the mid-cell, i.e. smaller rings were present in MOMP-labeled RBs that showed clear mid-cell constrictions (Fig 4a–4e and S3a–S3d Fig); 3) while ring diameter and ring thickness were variable (x, and z in Fig 4f), the apparent PG ring width (y, in Fig 4f) was relatively constant with an average of 138.6 ± 18.6 nm (n = 25); 4) for a subset of bacteria, mid-cell rings were entirely absent and instead replaced by small, full discs between two adjacent RBs, measuring 202.7 ± 24.2 nm in diameter at their widest points (n = 25, Fig 4c–4e and S3f–S3h Fig; arrows); and 5) instances of single RBs containing multiple PG rings and asymmetric localization of these rings between two/three cells apparently undergoing asymmetric division (Fig 4g–4k, S3e and S3f Fig).

### RBs take up and incorporate DAADs rapidly and processively during exponential growth

**Fig 5:**
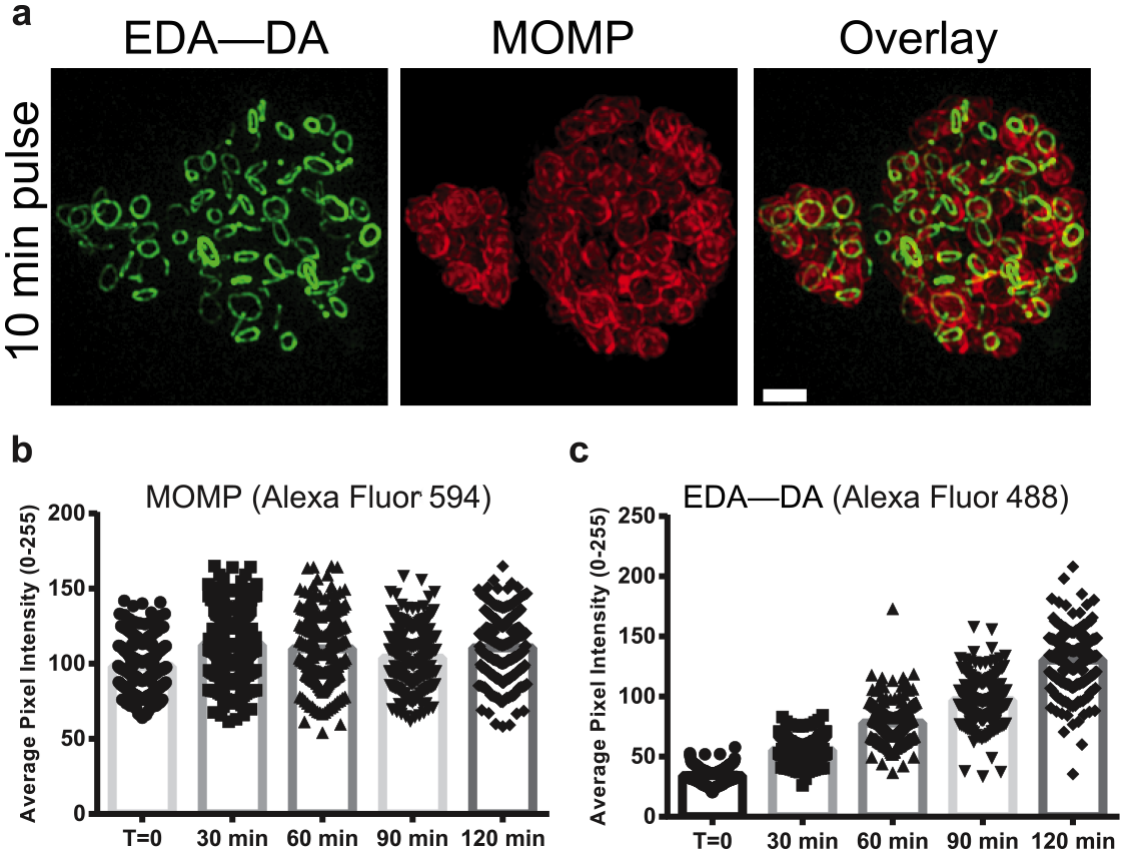
*C*. *trachomatis* incorporates DAADs rapidly during exponential growth. **(a)** SIM of chlamydial inclusions (18 hpi) incubated for 10 min with 4 mM EDA—DA. Z-stacks from chlamydial inclusions (18 hpi) incubated with EDA—DA were collected, maximum intensity projections constructed, and average pixel intensities calculated for entire inclusions for both MOMP **(b)** and EDA—DA **(c)** channels. ~200 inclusions were assigned average pixel intensities per time point measured and the entire experiment was carried out in biological duplicates. MOMP and PG labeling is as presented in Fig 2. Scale bar = 1 μm.

In rod-shaped bacteria, assembly of the complete division complex (and accompanying septal PG) occurs at the mid-cell just prior to the initiation of cell division, and only after a lengthy cell elongation phase[7]. Similarly, the model coccus-shaped organism, *Staphylococcus aureus*, undergoes a period of peripheral growth before onset of septal PG synthesis [33]. In contrast, although *C*. *trachomatis* strain L2/434 has a generation time of ~ 2.5 h to 3 h[34, 35], experiments with long DAAD incubation times (e.g. 18 h) revealed complete septal PG rings in the vast majority of RBs present within the developing inclusion (Fig 4a and 4b, S3 Fig). This suggests that synthesis and assembly of the septal PG begins unusually early in the cell cycle compared to other bacteria. In order to investigate the dynamics of PG ring formation, we varied the time that RBs were exposed to DAADs. Pulse labeling experiments on actively dividing RBs (18 hpi) confirmed that complete PG rings were detectable with as little as 10 min of DAAD labeling (Fig 5a), indicating that the basic PG ring assembly is also rapid. Although there was no significant change in the overall ring structure (i.e. a relatively constant ≤ 140 nm PG ring width) after labeling pulses longer than 10 min, the DAAD signal intensity gradually increased with increasing pulse duration. We later quantified this, allowing us to conduct the first kinetic analysis on the uptake and transport of any substrate by intracellular *Chlamydia*.

The close proximity of individual RBs within chlamydial inclusions and the difficulty in comparing fluorescence intensities of PG rings in dissimilar spatial orientations make single cell PG signal quantification practically impossible. Therefore, as a general quantitative approach, we tracked average epifluorescence values for individual inclusions using maximum intensity projections and the MOMP signal to normalize the average cell density per inclusion. We utilized an asynchronous infection model, allowing us to view a broad range of inclusion sizes (representing various stages of maturation / development) at each given time point. The range of inclusion sizes was similar for all time points, and the average inclusion size did not significantly differ between time points analyzed. Measurement of new DAAD incorporation over 2 h (from 18 to 20 hpi) showed that while mean MOMP fluorescence remained relatively constant (Fig 5b), mean PG fluorescence per inclusion gradually increased over the same time span (Fig 5c). No correlation was detected between inclusion size and average pixel intensity for either labeled PG or the major chlamydial outer membrane protein (MOMP) (S4a–S4c Fig), indicating that asynchronous chlamydial infections did not significantly affect the experimental outcome. Together, these results indicate that DAADs are continuously incorporated into one basic PG ring structure over several hours (Fig 5b and 5c) during the replicative phase of the chlamydial developmental cycle.

### Chlamydial PG ring assembly correlates with non-uniform localization of MreB patches

**Fig 6:**
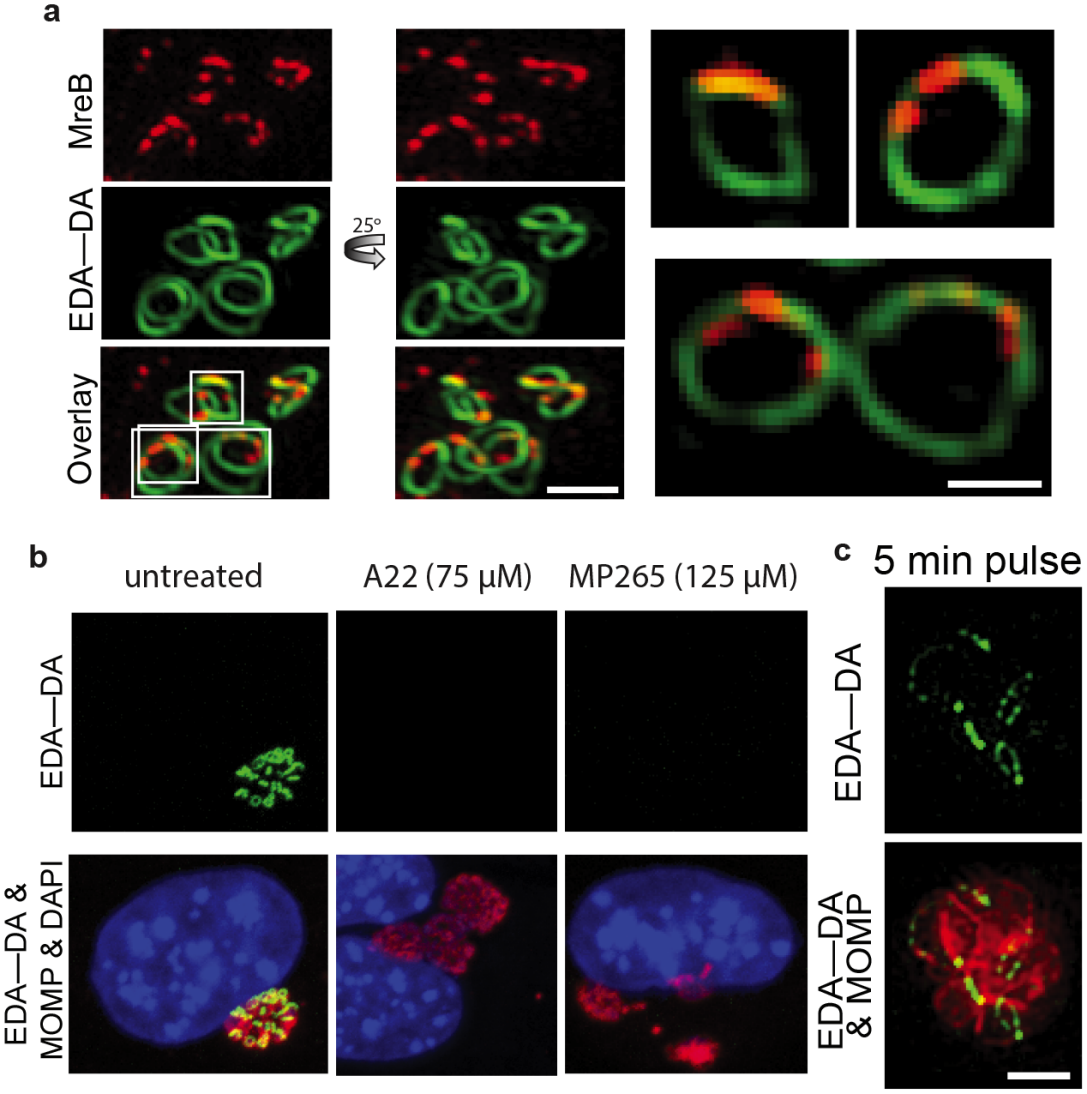
Chlamydial MreB is patchy, co-localizes to PG rings, and is required for chlamydial PG synthesis. **(a)** SIM of chlamydial PG localization in relation to polymerized MreB in inclusions 18 hpi incubated with EDA—DA for one hour. Right-most panels are magnifications of single imaging planes from **a**. **(b)** Chlamydial inclusions (18 hpi) grown in the presence of 4 mM EDA—DA and MreB inhibitors A22 or MP265 (added 12 hpi). MOMP and PG labeling is the same as in Fig 2. (**c**) SIM of EDA-DA labeled PG (green) and MOMP (red) within chlamydial inclusions (18 hpi) incubated with 4 mM EDA-DA for 5 minutes. Scale bars = 1 μm.

Rapid, yet persistent chlamydial PG ring assembly would require tight spatiotemporal coordination of new PG incorporation along the ring. We investigated the degree to which these mechanism(s) are coordinated by the chlamydial cell division machinery. The bacterial tubulin-like protein FtsZ plays an essential role in cell division by assembling the division apparatus at the correct division plane in almost all bacteria, chloroplasts, some mitochondria, and even some archea[7, 36–38]. Chlamydiae[9, 10] and its distant relative Planctomyces[39, 40] constitute the only two phyla of PG-containing bacteria that lack an FtsZ homolog[41]. Studies now suggest that in the absence of FtsZ, MreB may act as the division plane organizer in *Chlamydia*, since both MreB and the septal organizer RodZ localize to the division septum[42–44]. MreB is an actin-like protein that controls the cylindrical nature of rod-shaped bacteria by forming dynamic patches traveling around the circumference of the side walls. Since DAADs labeled chlamydial PG at the division septum, and chlamydial MreB has previously been shown to localize to the division septum [44], it is a logical candidate for facilitating PG ring assembly. By combining anti-chlamydial MreB antibody[44] with DAADs and 3D-SIM imaging, we found that in RBs chlamydial MreB is patchy, similar to the localization patterns reported in other bacteria [45–47], and that these MreB patches appeared to co-localize with PG rings (Fig 6a). Inhibition of filament formation with MreB depolymerizing agents, A22 and MP265[48, 49], resulted in a loss of MreB labeling in normal chlamydial RBs (S5a Fig), a reduction in the abundance of MreB patches in DCS-induced aberrant bodies (S5a Fig), and inhibition of DAAD incorporation (Fig 6b). No labeled MreB was visible in the metabolically inactive chlamydial EBs (S5b Fig) as previously reported[44]. These results strongly suggest that MreB polymerization is required for new PG incorporation along the septal PG ring structure in Chlamydiae.

Because MreB moves circumferentially along the lateral walls of many bacterial species [45–47], we hypothesized that MreB could be similarly dynamic along the chlamydial PG ring. We reasoned that we could infer MreB dynamics by labeling its downstream product, namely newly incorporated PG, with DAADs. Though indirect, a brief DAAD pulse would record the movement of MreB on the PG ring in the form of a fluorescent trace of new DAAD incorporation. A short (five minute) pulse with DAAD resulted in non-uniform, patchy PG trace distinguishable by epifluorescence (S6a Fig) and more clearly discernable at SIM resolution (Fig 6c). Anti-MreB signal partly co-localized with short EDA-DA pulses and the remaining signal was interspersed between PG patches, appearing to complement the newly forming PG arcs (S6b Fig). The lack of complete co-localization of MreB patches and PG patches indicates that we may lack the instrumental sensitivity to follow these rapid processes with ideal spatiotemporal resolution, however, these observations are consistent with the hypothesis that dynamic MreB patches facilitate incorporation of new PG along the septal plane, eventually completing a full PG ring in ~10 min (Fig 5a).

If A22 successfully blocks MreB-dependent PG incorporation in *Chlamydia*, we predict that inhibition of MreB polymerization should result in the accumulation of incomplete PG muropeptides, as is the case in *E*. *coli* [38]. Because classical analytical techniques[16, 17, 19, 21] are inadequate to detect chlamydial PG in lysates of infected cells, we employed a new, highly sensitive, immunodetection approach [50]. Chlamydial PG fragments are detected by the host cell via the intracellular NOD2 receptor, which recognizes N-acetylmuramic acid (MurNAc) present on muropeptide breakdown products of bacterial PG [50]. When coupled with mass spectrometry, this then can be used to measure the relative abundance of chlamydial PG muropeptides[50]. In order to determine if incomplete PG muropeptides accumulate when MreB polymerization is inhibited, we infected HeLa cells with *Chlamydia* for 18 hours followed by A22 (75 μM) treatment for 2 hours. Lysates from infected cells treated with A22 resulted in a significant increase in NOD2 signaling when compared to untreated (no A22) lysates from *Chlamydia*-infected cells (S7a Fig).

Analysis of A22-treated and untreated cell lysates by Mass and Enhanced Product Ion (EPI) scans revealed an increase in products of ~477, 494, and 666 mass units (mu) (S7b and S7c Fig). These peaks correspond to a degradation product of chlamydial muramyl dipeptide (MDP, S7d Fig) and the free species of chlamydial MDP and MTP, respectively [50]. Subsequent analysis via tandem mass spectrometry (MS/MS) confirmed that the breakdown products of the 477 mu peak were identical to those of an MDP fragment from *C*. *trachomatis* (S7d Fig) and column retention times for 494, 666, 653 peaks correspond to those observed previously for chlamydial MDP, MTP, and the cross-linked PG fragment [50]. In addition, we found that the relative abundance of a cross-linked, PG degradation product of 653 mu (described previously, [50]) decreased significantly in A22-treated samples (S7c Fig). As the structures for the UDP- precursors of chlamydial lipid I synthesis have not yet been identified / characterized, we cannot definitely state whether the accumulation of MDP and MTP in A22-treated cells is the result of PG degradation, incomplete lipid I synthesis, or both. In an attempt to ascertain whether these fluctuations in muropeptide abundance resulting from A22-treatment were similar to those seen when PG biosynthesis is directly inhibited, we examined samples collected from *Chlamydia*-infected cells that had been treated with DCS, a D-Ala-D-Ala ligase inhibitor that arrests PG biosynthesis (Fig 1). We found that, similar to A22, DCS treatment resulted in an increase in the abundance of both chlamydial MDP and MTP and a decrease in 653 muropeptide species (S7c Fig).

### MreB is essential for chlamydial replication, differentiation, and growth

In addition to blocking PG biosynthesis, inhibition of chlamydial MreB polymerization also affected *Chlamydia* replication and its interaction with host cells throughout its biphasic life cycle. Consistent with previous work [43, 51], we found that A22 inhibition of MreB after the EB to RB transition (8 hpi) resulted in arrest of RB replication (S8a and S8b Fig). A22-treated RBs also became slightly enlarged, though significantly less than aberrant bodies induced by exposure to antibiotics targeting various penicillin-binding proteins (PBPs), i.e. β-lactams[43]. Unlike β-lactam induced aberrant bodies that still show strong DAAD labeling [9], A22-induced aberrant bodies completely lacked detectable PG (Fig 6b). This observation suggests an essential role for MreB as well as PG in maintaining chlamydial cell size and shape.

When infected cells were treated with MreB inhibitors prior to the EB-RB transition (< 8 hpi), some aberrant body formation (~ 4–6%) was observable, but the vast majority of bacteria appeared to remain in an EB-like state of development, despite being intracellular for more than 16 hpi (S8b, S8c and S8f Fig). Inhibition of the normal developmental cycle is reversible as these EB-sized *Chlamydia* appeared to differentiate into RB sized *Chlamydia* when the inhibitor was removed (S8b and S8f Fig). This suggests that in addition to maintaining cell size and division, functional MreB may also be critical for the EB to RB transition. In order to establish how compounds A22 and MP265 affect the ability of *Chlamydia* to complete its developmental cycle, infected cells were treated for the indicated times with each compound, and EBs were collected at the termination of a normal chlamydial developmental cycle (44 hpi). Numbers of inclusion forming units (IFUs) were then obtained by re-infecting monolayers to establish the number of viable EBs present in each test group. We found that when *Chlamydia*-infected cells were treated with either A22 (75 μM) nor MP265 (125 μM) for brief (one hour) periods, the number of IFUs recovered was only slightly fewer than in our untreated control groups (S8d Fig). This was the case when compounds were added early in infection (2 hpi) when *Chlamydia* is in the EB developmental state or later in infection (18 hpi) when EBs have all transitioned to replicative RBs **(compare UTD**, **2–3 hpi and 18–19 hpi columns**, S8d Fig). When compounds are added to infected cells 2 hpi or 18 hpi and left in the growth medium for the remainder of the developmental cycle (42 and 26 hours, respectively) few if any IFUs are recoverable (**2–44 hpi and 18–44 hpi columns,** S8d Fig), as has been reported previously [43]. However, if compounds are added 2 hpi and removed after 8 hours (at 10 hpi) viable EBs are recoverable when harvested at 44 hpi **(2–10 hpi (44 hpi) columns**, S8d Fig). In the A22-treated group, ~10^4^ IFUs were recovered, whereas in the MP265-treated group ~10^7^ IFUs were recovered. Compound A22 has previously been reported to exhibit off-target effects in other bacterial systems [49], and we suspect that this may explain the difference in IFUs recovered between A22 and MP265 treatment groups. When IFUs were collected at 52 hpi (as opposed to 44 hpi), the number of recovered IFUs increased substantially (**2–10 hpi (52 hpi) column**, S8d Fig). By contrast, no similar increase in IFUs recovered was observed in MP265-treated groups that were allowed an additional 8 hours of recovery time, prior to EB harvesting.

Consistent with the idea that polymerized MreB is a general facilitator of chlamydial growth and the biphasic developmental cycle, MreB inhibition also arrested inclusion formation/maturation as evidenced by the lack of discernable inclusion membrane protein A (IncA) labeling in the inclusion membrane upon A22 treatment, while DCS-treated bacteria still developed mature inclusions (S8e Fig). Time course studies revealed that inclusion maturation continued to be suppressed when the MreB polymerization inhibitor was added as late as 12 hpi, approximately four hours after the EB-RB transition is known to occur [52] and ~2–4 hours before IncA can be detected at the inclusion membrane. In contrast, DCS-treated *Chlamydia* were capable of expressing IncA and incorporating it on the surface of their inclusions. When the inhibitor was removed as late as 10 hpi, chlamydial inclusions resumed normal maturation as evidenced by the appearance of IncA at the inclusion membrane with no aberrant bodies visible at 22 h (S8f Fig). Taken together, these data suggest that MreB is a key regulator of chlamydial growth and development essential for the EB to RB transition (~8 hpi), cell enlargement and division, PG biosynthesis in RBs (>8 hpi), maturation of inclusions (>12 hpi), and differentiation of RBs back into EBs.

### Nascent chlamydial PG assembly is D,D-transpeptidation/carboxypeptidation independent

DAAD incorporation into chlamydial PG is independent of the D,D-transpeptidation/carboxypeptidation activity of PBPs, as evidenced by the persistent and rapid labeling in ampicillin-treated aberrant bodies[9]. In the presence of β-lactam antibiotics (ampicillin or piperacillin) the PG signal followed irregular branches within the enlarged, chlamydial aberrant bodies and rarely exhibited complete ring morphology (S9a and S9b Fig). Despite this abnormal localization, the PG remained susceptible to lysozyme, an enzyme that targets 1,4 β-linkages in the glycan polymer chain (S9c Fig). Consistent with the link between chlamydial MreB and PG synthesis described above, the MreB signal co-localized along these PG branches as individual patches and, in some instances, extended structures (S9d Fig). We also observed similarly patchy localization of MreB in DCS-induced aberrant bodies (S9e Fig). These results, taken together, suggest that chlamydial MreB can still facilitate the formation of transglycosylated strings of nascent PG in the absence of D,D-transpeptidation/carboxypeptidation activity and an increase in cell size, but that PG cross-links are essential for the regulation of cell size and the initiation of cell division.

### Chlamydial PG rings lose DAAD signal evenly and slowly

Our data strongly suggest that chlamydial MreB (in combination with PBPs) is responsible for the assembly of a properly sized PG ring localized at mid-cell and the division septum in actively dividing bacteria. However, these data do not explain how PG rings follow the cell constriction. A degradation and/or reshaping mechanism must be acting on this structure, as we observe rings of different diameters but not a full division plane until the very end of cell division (Fig 4 and S3 Fig). Based on our observation that the chlamydial PG biosynthesis machinery rapidly synthesizes and maintains a defined PG ring structure along the constriction plane, we reasoned that the older PG might be degraded homogenously around the ring as new PG is synthesized; we will refer to this as the ‘homogeneous model’. Alternatively, new PG could be synthesized from the center of each PG ring band and push old PG outward towards the cell poles, as occurs in PG elongation of cocci and oval species, and be degraded at the edge of the DAAD-labeled ring band thereby restricting PG to the mid-cell (S10a Fig). For simplicity, we will refer to this putative mechanism as the ‘bidirectional model’.

**Fig 7:**
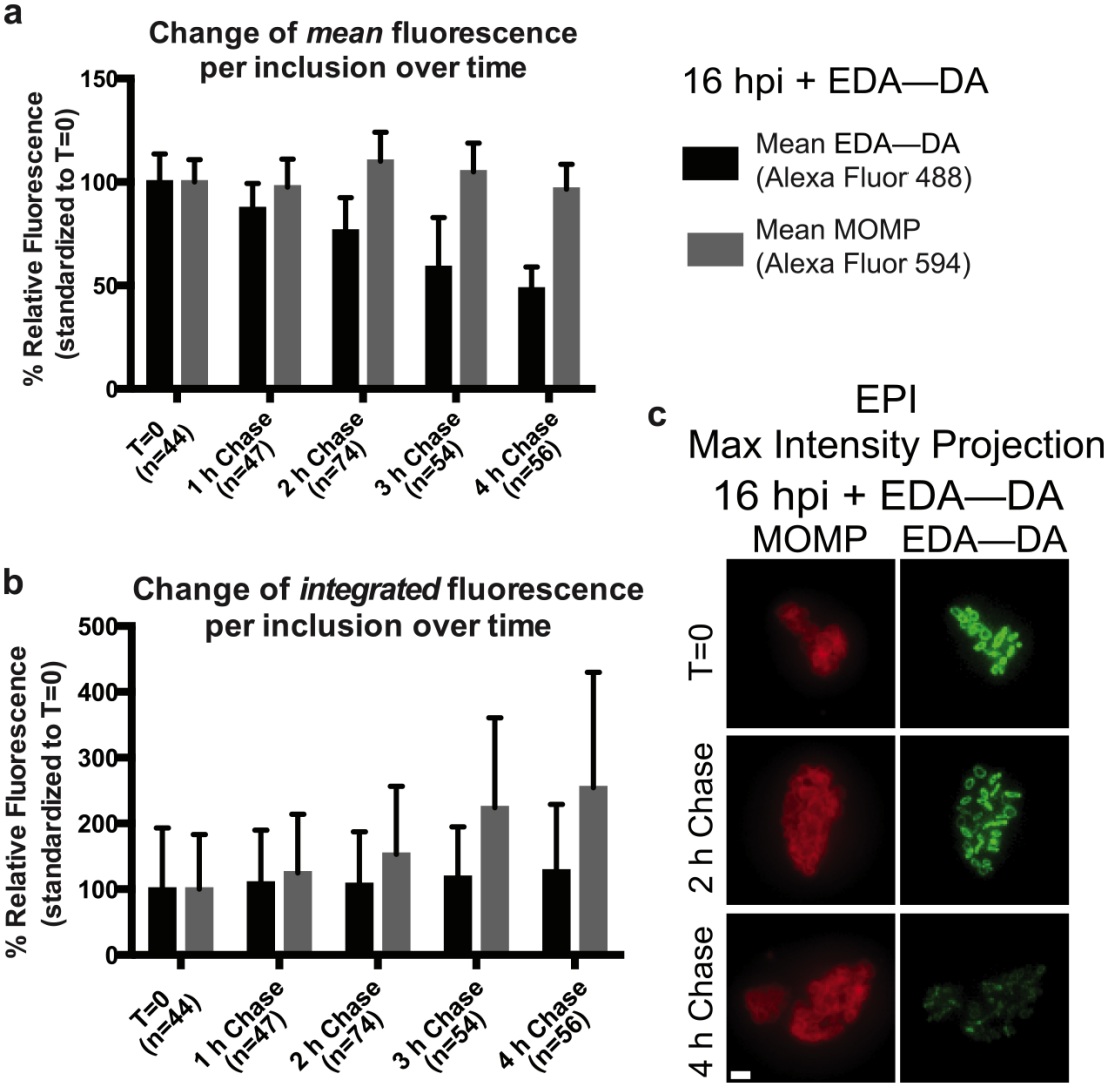
Pulse-chase experiments establish preliminary kinetics of PG disassembly in chlamydial RBs. Change in mean fluorescence **(a)** and integrated fluorescence **(b)** per inclusion over time from chlamydial inclusions pulsed 1 h with EDA—DA after which the medium was removed and replaced with probe-free medium. Subsequent measurements were taken at indicated time points (i.e. chase). **(c)** Representative epifluorescence (EPI) maximum intensity projections of labeled chlamydial inclusions after the initial pulse and successive chases are shown. Scale bar = 1 μm.

To explore this reshaping mechanism, we first measured how PG rings age. *Chlamydia* inclusions (18 hpi) were labeled with a DAAD for one hour (pulse) after which cells were washed in fresh medium and then allowed to incubate in medium without probe (chase). Loss of PG fluorescence signal from labeled chlamydial inclusions over time was then quantified following appropriate controls similar to earlier experiments (S10b and S10c Fig). Average fluorescence pixel intensities of whole inclusions, calculated over a four hour chase, indicated that the mean PG signal per inclusion (Average [Total PG signal per inclusion / area of inclusion]) decreased by 50% over approximately three hours, while the mean cell density (represented by Average [Total MOMP signal per inclusion / area of inclusion]) did not appear to change (Fig 7a and 7c). In contrast, a complementary analysis of the same data set showed that while the average integrated MOMP signal per inclusion (Average [Total MOMP signal per inclusion]) steadily increased (due to an increase in the number of cells per inclusion during chases), the average integrated PG signals per inclusion (Average [Total PG signal per inclusion]) did not change (Fig 7b and 7c). This suggests that the pulsed DAAD within an inclusion is distributed between daughter cells during the chase, though it is not currently possible to ascertain whether all of the free DAAD is fully incorporated into chlamydial PG during the hour-long pulse. Some amount of unincorporated DAAD may be simply retained by replicating RBs and incorporated into newly synthesized PG at later time points.

Although the time required for mean PG signal reduction by 50% is close to the estimated doubling time of *C*. *trachomatis* strain L2/434 (2.5–3 hours)[52], the overall thickness of the rings did not appear to change with longer chase times, and PG ring width (y, in Fig 4f) remained relatively constant with an average width of ~156.9 nm (sd 12.8 nm; n = 10) at t = 1 h, an average of 147.9 nm (sd 20.7 nm; n = 19) at t = 3 h and an average of 155.9 nm (sd 18.1 nm; n = 18) at t = 4 h, compared to the aforementioned 138.6 nm ± 18.6 nm at t = 0. Inherent to the design of our pulse-chase experiment, if a bidirectional mechanism existed, older DAAD signal would split in two and become thinner at the edges as unlabeled new PG is incorporated in the middle (S10a Fig). However, as we are currently operating at the limits of 3D structured illumination microscopy, future studies conducted at higher resolutions will be needed to definitely refute the bidirectional synthesis model.

### Inhibition of MreB-dependent PG synthesis leads to rapid PG degradation

We previously showed that DAADs can substitute for natural DA—DA in *C*. *trachomatis*[9] and that aberrant bodies induced by β-lactam antibiotic inhibition of chlamydial periplasmic D,D-carboxypeptidases/transpeptidases (i.e. PBPs) are still capable of incorporating DAADs ([9], and S9a, S9b and S9d Fig). These results demonstrate that chlamydial PBP2 and PBP3, which are both inhibited by ampicillin, are not required for DAAD incorporation and that incorporation of label into chlamydial PG likely occurs in the cytoplasm. The slow decrease of the PG signal and the distribution of the signal between the newly formed daughter cells observed during pulse-chase experiments (Fig 7a and 7b) could therefore be due to incorporation of DAADs trapped within the inclusions and/or the cytoplasm of chlamydial RBs. This continuous DAAD incorporation could obscure the true kinetics of the observed PG degradation mechanism. We reasoned that if incorporation of new material was arrested by inhibition of MreB, we could uncouple chlamydial PG synthesis from degradation.

**Fig 8:**
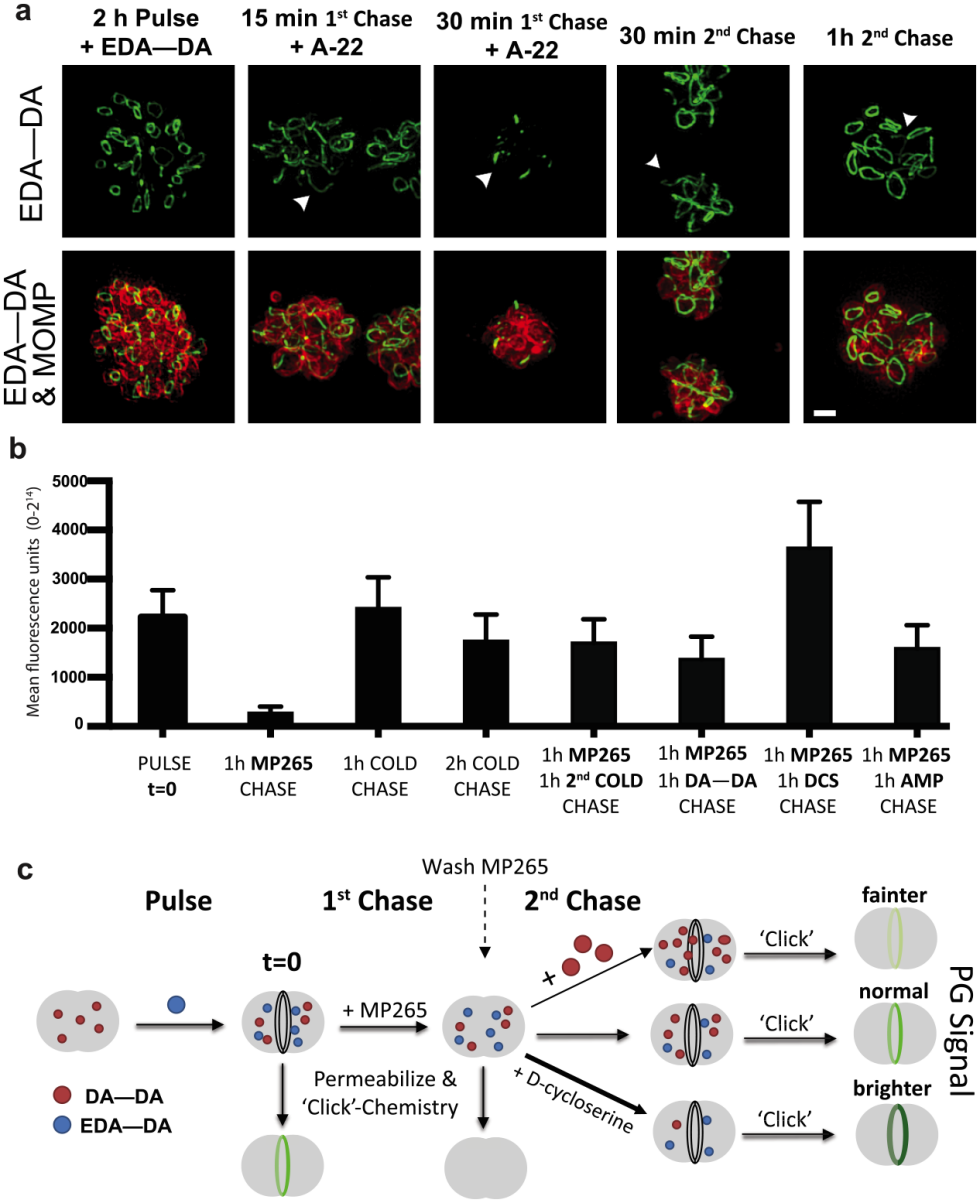
**(a)** SIM of chlamydial inclusions in which cells were pulsed for two hours with 4 mM EDA—DA, after which the MreB polymerization inhibitor A22 (75 μM) was added to the medium. In the two far-right panels, medium containing A22 was removed after one hour. Cells were then washed, fresh medium without new EDA—DA was added, and cells were allowed to grow an additional 30 and 60 minutes, respectively. At indicated time points, cells were fixed, permeabilized, and fluorescently labeled via click chemistry and anti-_CT_MOMP. Scale bars = 1 μm. **(b)** Quantitative analysis of the mean fluorescence intensity of chlamydial inclusions initially pulsed with EDA-DA and subsequently chased under the times and conditions indicated. Fluorescence values were collected for ~250 inclusions per group, statistics were obtained utilizing an unpaired t test with Welch's correction and error bars represent standard deviations of the mean. Analysis was conducted in triplicate and is representative of two biological replicate experiments. **(c)** Graphical model representing the experimental approach and results from **(b)**.

When RBs (18 hpi) were pulsed with DAADs and chased with fresh medium containing A22, the signal intensity of labeled PG rings began decreasing in a non-uniform manner within 15 minutes (Fig 8a) with no significant change in PG ring width, with an average width of 151.7 nm (sd 19.9 nm; n = 17) at t = 15 min, an average of 153.9 nm (sd 16.4 nm; n = 17) at t = 30 min and an average of 142.2 nm (sd 19.6 nm; n = 9) at t = 45 min, compared to 156.7 nm (sd 17.2 nm; n = 9) at t = 0. At 30 minutes, all PG rings were almost completely dissociated with only punctate labeling present on a handful of RBs within any given inclusion. After one hour of A22 or MP265 treatment, no labeling of chlamydial PG was discernable (A22 / MP265 chase) compared to the control (first chase) (Fig 8b). Taken together, our data indicate the presence of two separable and highly active mechanisms critical for the reshaping of the chlamydial PG ring as RBs constrict during cell division: MreB-dependent PG synthesis and an as yet uncharacterized degradation mechanism(s).

### *Chlamydia* accumulates and retains D-amino acid dipeptides

When cells were pulsed with DAADs and chased with fresh medium containing A22 (resulting in complete loss of PG signal), subsequent removal of A22 and cell recovery in fresh medium (in the absence of exogenously added DAAD or A22, second chase), resulted in a striking restoration of PG ring signal in chlamydial RBs (Fig 8a and 8b). This is indicative of relatively high concentrations of free (and/or cytoplasmic PG precursor-bound) DAADs being retained by the chlamydial cells/inclusions, despite media changes and wash steps subsequent to pulse-labeling. Since MreB inhibition is reversible (S8 Fig), washing out A22 is expected to restart new PG synthesis and also incorporation of any residual DAADs remaining within cells/inclusions. However, when we conducted the second chase in the presence of very high concentrations of the native unlabeled dipeptide DA—DA (second DA—DA chase), we observed a slight drop in PG signal recovery compared to when no native DAAD was added (Fig 8b and 8c) which may indicate that the extra DA-DA is competing with the unincorporated probe during the recovery. In contrast, when the second chase was done in the presence of DCS, an inhibitor of DA—DA synthesis, we observed an increase in PG signal recovery (Fig 8b and 8c). This was expected, as depleting the cellular pool of the competing, native DA—DA would increase the frequency of DAAD incorporation. When the second chase was conducted in the presence of ampicillin, we observed only a slight decrease in signal recovery compared to the second cold chase (Fig 8b and 8c), indicating that probe PG incorporation (S9a and S9b Fig) and degradation are independent of D,D-transpeptidation/D,D-carboxypeptidation activity, which are both inhibited by β-lactam antibiotics.

The competition of the native DA-DA with DAADs is also evident in the decrease in EDA-DA labeling when our initial EDA-DA pulse is followed by a native DA-DA chase (S11a Fig). We observed an even greater reduction of alkyne containing EDA-DA labeling when we chase with the alternative azide containing DAAD, ADA-DA (S11b Fig), however, this reduction in fluorescence is most likely the result of these two PG incorporated DAADs within close proximity forming stable conjugates with each other via azide-alkyne [3+2] cycloaddition (and not their corresponding fluorophores) upon the initiation of the click chemistry reaction. Nevertheless, the retention of components of immunogenic PG (DAADs), the absence of PG biosynthesis / retention in the extracellular form of *Chlamydia*, and the limiting of PG to a mid-cell ring often localizing to the division septum of its replicative form, all indicate that *Chlamydia* restricts its PG to where and when it is absolutely needed. In the context of an obligate, intracellular pathogen that has co-evolved with vertebrate hosts for hundreds of millions of years, this severe limitation on PG biosynthesis and maintenance is indicative of pathoadaptation by *Chlamydia*.

## Discussion

To better understand the role of PG in the life cycle of obligate, intracellular pathogenic *Chlamydia*, we characterized the assembly and maintenance of this critical cell wall component. Our observations show that four species of pathogenic C*hlamydia* maintain a distinctive PG ring limited to a small region at mid-cell and localizing to the septum in bacteria actively undergoing division. We refer to this structure as a unique and limited PG ring based on four major observations:

1. The chlamydial cell maintains a narrow, dynamic PG ring structure restricted to the apparent division plane of the bacterium.
2. PG is found only in the actively growing replicative (RB) form of *Chlamydia*.
3. This limited PG is a common and unique feature of the pathogenic Chlamydiaceae family.
4. Assembly and maintenance of this limited PG ring is controlled by MreB in conjunction with PBPs and a yet-unknown PG turnover mechanism.

Based on current detection techniques and data from previous publications, we conclude that these PG rings constitute the only PG cell wall structure in actively growing pathogenic *Chlamydia*. Regardless of the duration of labeling with DAADs, rings of relatively constant width represent the sole fluorescence trace of PG observed in the replicative form of pathogenic *Chlamydia*, as opposed to the uniform, peripheral labeling present in other bacteria with classical, cell-encompassing PG sacculi following prolonged labeling[9]. While our experimental approach limits labeling of PG to the fourth amino acid of the stem peptide, we surmise that position 4 is very stable, as no L,D transpeptidases, which would cleave the stem peptide at this position, appear to be encoded within the chlamydial genome[23]. Our observations also match immunofluorescence data obtained by researchers utilizing antisera raised primarily against mycobacterial PG, which gave structures very similar to the DAAD-labeled PG rings present exclusively in RBs[32]. Pathogenic *Chlamydia* lack a peripheral peptidoglycan layer when imaged via electron microscopy[13], and sacculi have never been successfully purified from chlamydial cells[16, 19]. The existence of a thin, lysozyme sensitive ([9] and this work), MurNAc-containing [50] PG ring present only in RBs would account for the findings of past researchers, who each reported finding only trace evidence, if any, of PG in *Chlamydia* [14, 15, 18, 20, 32]. Additionally, the absence of PG labeling in EBs is consistent with the lack of NOD1 and NOD2 stimulatory PG fragments in lysates of chlamydial EBs, with only trace amounts of the PG-specific sugar, MurNAc, detectable in infected cell lysates after the EB-RB transition [50].

Our limited PG model is also supported by evolutionary analysis. We observed narrow PG ring structures in four different pathogenic chlamydial species that are evolutionarily representative of the *Chlamydia* genus. In contrast, Pilhofer *et al*. reported that *Parachlamydia* endosymbionts (environmental *Chlamydia*-like bacteria represented by *P*. *amoebophila*) possess a conventional, structurally supportive, shape-determining sacculus[10]. *Simkania negevensis*, a pathogenic *Chlamydia*-like bacterium associated with community-acquired pneumonia in humans[6, 53], does not appear to possess a PG sacculus. This may hint at a speciation event of ancestral Chlamydiae: the pathoadaptation of ancestral *Chlamydia* (possibly also *Simkania*) to their human/animal hosts may have resulted in spatially restricting PG to a bare minimum, a small ring, and temporally limiting the period of PG synthesis to actively replicating, intracellular RBs. Future studies examining the PG of other environmental and pathogenic *Chlamydia* species will shed light on the prevalence of this peculiar PG architecture throughout the Chlamydiae.

We propose that pathogenic *Chlamydia* do not require a sacculus due to the osmotically stable environment in which they reside. It is noteworthy that *E*. *coli*, upon treatment with penicillin-based antibiotics under osmotically stable conditions does not lyse, and instead continues to expand, unable to divide (similar to ampicillin-induced, chlamydial aberrant bodies). The genomes of some obligate extracellular (*Mycoplasma*) and intracellular (*Ehrlichia*, *anaplasma*) pathogens lack all or the vast majority of PG synthesis genes, indicating that PG (and by extension, a sacculus) is dispensable for the survival of obligate extracellular / intracellular pathogens. While this line of reasoning does not account for the presence of sacculi in some obligate intracellular pathogens such as *Coxiella*, these exceptions can often be explained when their various environments and lifecycles are considered. Rickettsiae, for example, are highly pleiomorphic organisms and generally have broad host ranges that include arthropods, potentially subjecting them to less osmotically stable conditions than those found within vertebrate hosts. *Coxiella* in particular is among the most resistant of the Rickettsiae to adverse environmental conditions (i.e. osmotic stress) indicating that one major function of a PG sacculus (osmotic protection) potentially remains essential to the organism.

Assuming that *Chlamydia* has effectively dispensed with the need to withstand osmotic stresses by adapting to an obligate, intracellular niche, we speculate that PG would still be maintained by the organism i) if it was essential for cell division, and/or ii) if it acts as a signaling molecule to other microbes or to the host immune system, to the direct benefit of *Chlamydia*. Limiting, masking, or removing PG (and other immunostimulatory PAMPS) is likely an intrinsic adaptation of ancient, obligate intracellular pathogens. Such a reduction of its immunogenic profile is beneficial for a pathogen so long as this does not compromise core functions essential for its survival. As a logical extension of this model, the complete removal of PG from the organism would be ideal. However, while other pathogens (such as *Mycoplasma*) have developed cell division mechanisms that function in the absence of PG, *Chlamydia* has not. We propose that by limiting PG to the septum during its replicative growth phase, *Chlamydia* maintains a minimal PG synthesis activity essential for division while also minimizing its recognition by host innate immune receptors. PG fragments are potent signaling ligands for both NOD1 and NOD2 receptors of the innate immune system[54], and limiting their abundance may significantly mitigate *Chlamydia’s* immunogenic profile. This hypothesis is supported by our finding that DAAD probes, unnatural substitutes for the PG D-Ala-D-Ala residue [9], appear to be retained within RBs and distributed between the daughter RBs upon division. The recent discovery [50] that pathogenic *Chlamydia* incorporates glycine into the first position of the PG stem peptide further supports pathoadaptation by the microbe. This specific modification of the peptide chain has only been observed in the intracellular pathogen *Mycobacterium leprae*[55] and studies have shown that alterations at this amino acid position can decrease immunoadjuvant capacity[56] as well as significantly affect MDP recognition by the NOD2 receptor[57].

**Fig 9:**
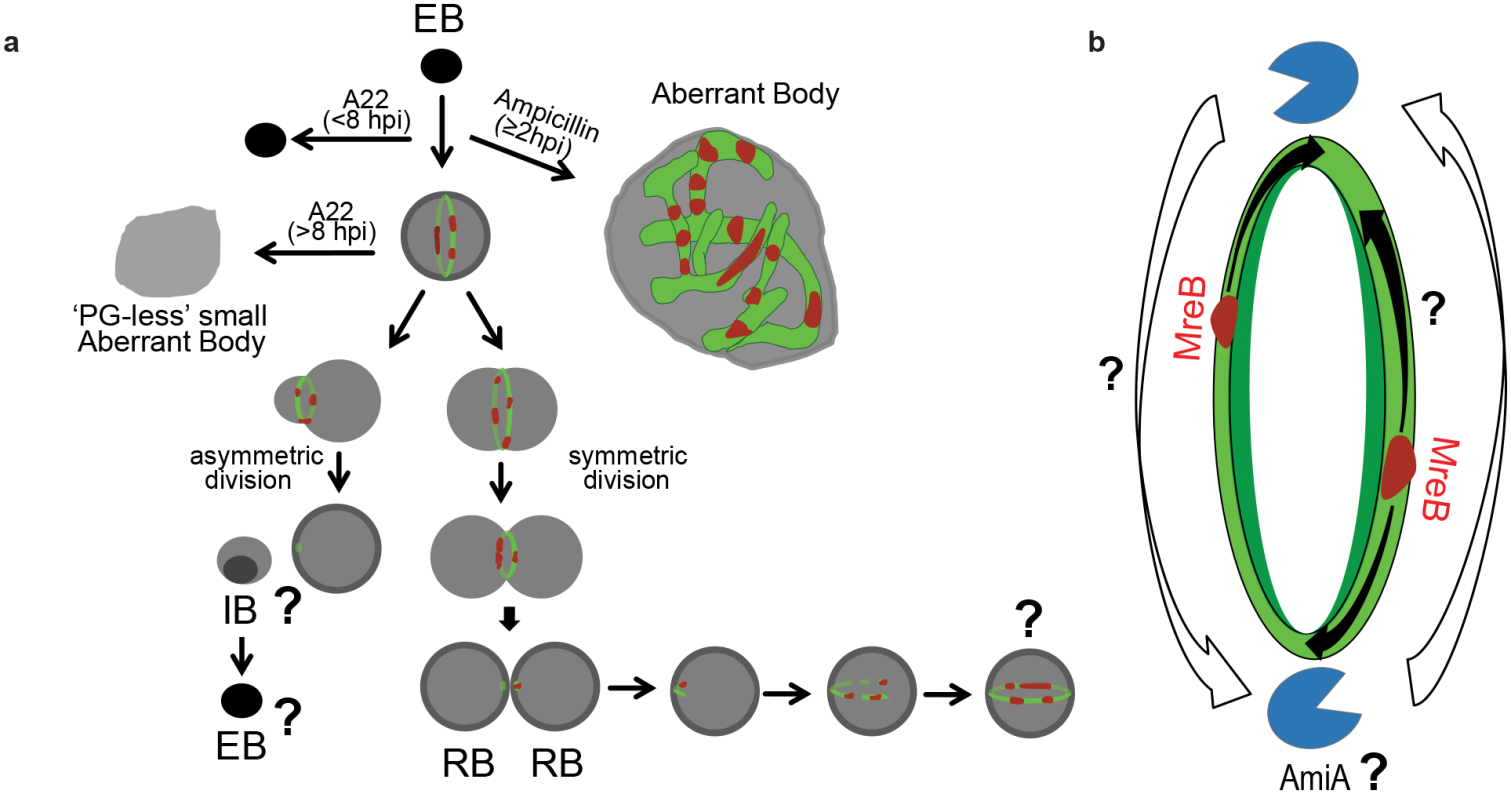
Proposed model for PG biosynthesis and maintenance in pathogenic *Chlamydia*. **(a)** Upon invading a host cell, MreB facilitates mid-cell PG (green) ring formation 8 hpi and the first cell division occurs. Inhibition of MreB (red) prior to EB-RB transition prevents this first division and results in accumulation of intracellular ‘EB-like’ particles in the host cytosol. Once RBs form, PG is constantly synthesized and incorporated into the ring non-uniformly by dynamic MreB patches. If MreB polymerization is inhibited, the PG ring dissociates, resulting in slightly enlarged, static, PG-less aberrant bodies. Inhibition of PG crosslinking by β-lactams does not affect transglycosylase activity and MreB localization, but instead results in unchecked enlargement of the RB. Bottom, upon cell division, the new division planes form immediately and perpendicular to the previous division plane. During transition to non-replicative EBs, MreB and PG biosynthesis enzymes are downregulated [58], resulting in the complete disassembly of the PG ring. **(b)** Chlamydial PG biosynthesis and degradation mechanisms overlap and are active throughout the cell cycle. MreB (red) moves along the ring plane (black arrows), initiating the non-uniform synthesis of new PG (green) and influencing the action of an unknown degradation mechanism that continuously removes older material (white arrows). PG degradation enzymes (amidases and lytic transglycosylases) are visualized as blue pacmen.

Our observations indicate that chlamydial PG rings exclusively localize to the apparent division plane of RBs. Upon the onset of division, the ring contracts with the outer membrane and immediately upon division PG appears to localize in polar patches. PG is only ever discernable in these polar patches or complete rings, which suggests that the new chlamydial division plane forms immediately after the previous division. These polar PG patches may act as cues for priming the new division plane, which would force it to form perpendicular to the previous one (Fig 9a), similar to what occurs in other cocci [33]. Complete PG rings are also discernable prior to any apparent sign of constriction (Fig 4 and S3 Fig). This observation may indicate that the chlamydial PG ring either must first mature before driving constriction or it may confer some additional utility to the organism, such as structural support or acting as a scaffold for the division and membrane constriction machinery. Additionally, while cell division following ring constriction most often occurs symmetrically, the occurrence of bacteria with asymmetric division planes may serve to normalize cell size or to initiate the RB-EB transition.

The formation of incomplete PG rings upon short DAAD pulses, the patchy, partial colocalization of MreB on newly forming PG patches, the requirement for a functional MreB in order to synthesize and assemble PG rings, and the patchy and dynamic nature of MreB in other bacteria[45–47] all support a model in which new PG rings expand from polar patches with the aid of a mobile MreB upon completion of a cell division event (Fig 9a). The tendency of newly incorporated PG signal trailing the MreB patches might be due to the incomplete saturation of DA-DA pools with our modified dipeptides. Alternatively, there might also be a threshold of DAAD-labeled PG subunits that need to be built into the edge(s) of growing PG arcs by the pioneering MreB before it can be detected by our imaging conditions. Either would prevent us from unambiguously visualizing complete colocalization of MreB patches with short pulse PG patches. Therefore we suspect that these process may occur too rapidly for us to accurately visualize with technology currently available. We speculate that the MreB-facilitated PG synthesis occurs in a directional manner around the septal ring, constantly and dynamically threading the new PG into the older, ring-shaped material. While MreB co-localization with PG strands in enlarged aberrant bodies upon β-lactam inhibition of PBPs is consistent with this model, this observation also shows that transglycosylation in *Chlamydia* can be uncoupled from D,D-transpeptidation/carboxypeptidation. D,D-transpeptidation/carboxypeptidation may still play a role in defining the proper ring shape for division, but not for nascent PG assembly. We believe that future studies utilizing fluorescent protein fusions of relevant chlamydial proteins, such as MreB, coupled with live-cell optimized PG labeling DAADs will allow for these models to be tested in real-time via time-lapse microscopy.

We showed that inhibition of MreB arrests the EB to RB transition early in infection, the growth of RBs and the maturation of chlamydial inclusions at mid-infection, and the RB to EB transition late in infection. All of these phenotypes are reversible when the MreB inhibitor is removed. Therefore, we conclude that MreB is required not just for chlamydial replication, but also for cell differentiation and growth. There is a distinctive association between the spatiotemporal distribution of MreB, PG synthesis, and chlamydial cell growth. MreB and PG are both absent from chlamydial EBs and aberrant bodies induced by MreB inhibition. PG biosynthesis and cell division genes are both up-regulated upon initiation of the EB to RB transition[58] and we have shown that MreB localization and PG biosynthesis occur only after the transition. MreB and PG are both present and co-localize in normally growing RBs as well as in β-lactam induced aberrant bodies, which unlike aberrant bodies that form due to MreB inhibition, continue to grow at the same rate as healthy cells[59] while exhibiting long strands of nascent PG and patchy MreB (S9 Fig). Similar cell enlargement occurs in DCS-induced aberrant bodies that appear to be able to assemble an uncross-linkable, yet still polymerized nascent PG [32]. DCS treatment results in the accumulation of degraded muropeptides and/or PG precursors in *Chlamydia* and incomplete muropeptides have been shown to incorporate into the PG of *E*. *coli*, following DCS treatment [60]. Our observations, as well as those of others[32] indicate that PG glycan chains in ampicillin and DCS-induced aberrant bodies still form, giving weight to the hypothesis that nascent PG biosynthesis is independent of cross-linked PG. In the case of DCS-treated cells, incomplete muropeptides can still transit to the periplasm and incorporate into newly forming PG glycan chains, but cannot form proper crosslinks[60]. This is in contrast to inhibition of MreB function in chlamydial RBs, which leads to arrest of cell growth and absence of PG synthesis. RBs lacking only D,D-transpeptidation/carboxypeptidation activity (inhibited either directly by β-lactams or indirectly by DCS), continue to enlarge, synthesize and assemble nascent PG, but appear to lack the ability to regulate their size, likely due to poor coordination of proper cell division and the absence of cross-linked PG. This also indicates that chlamydial PG may have a cellular organizational role other than simply facilitating cell division.

While our initial observations indicated chlamydial PG was comprised of relatively fixed and static rings[9], this PG ring has proven to be highly and uniquely dynamic, as evidenced by its rapid assembly and, in the absence of functional MreB, disassembly. Other bacteria that grow from the mid-cell synthesize new PG at the septum, splitting the older septal PG and pushing it outwards toward the poles where it is eventually modified and/or degraded. Given that *Chlamydia* maintain PG as a ≤ 140 nm ring, we are currently unable to test this bidirectional model of PG reshaping in *Chlamydia* (S10a Fig), as the width of the PG rings is currently at the resolution limits of SIM imaging systems. Assuming that A22 dispersion of MreB polymers does not significantly affect the activity of the PG degradation mechanism, we favor a model in which, as the cell grows and divides, the PG ring in pathogenic *Chlamydia* is constantly re-sculpted by coordinated, yet independent PG synthesis and degradation mechanisms (Fig 9b).

Our model for the maintenance of chlamydial PG rings predicts the presence of an equally dynamic and closely localized PG degradation mechanism accompanying MreB-facilitated PG synthesis (Fig 9b). This degradation mechanism would likely include a yet uncharacterized lytic transglycosylase capable of cleaving the PG glycan chains and the chlamydial amidase, AmiA [61, 62], which cleaves the peptide stem from the N-acetylmuramic acid. Interestingly, the chlamydial *amiA* is expressed simultaneously with genes of the PG biosynthetic pathway[58] and the protein localizes diffusely in the periplasm[51]. The amidase encoded by *C*. *pneumoniae* possesses carboxypeptidase activity and lacks the regulatory domain present in the homologous *E*. *coli* protein[61]. A diffuse and constitutively active chlamydial AmiA possessing both amidase and carboxypeptidase activities could explain how a PG ring of constant width is maintained in normal RBs and how aberrant PG forms in β-lactam-induced aberrant bodies. When MreB is not functional, a diffuse and active AmiA would completely degrade older PG with no new PG to take its place. This could also explain the slight enlargement of RBs in the presence of A-22; in the absence of a strong PG belt, the RBs might expand slightly to reach the hydrostatic equilibrium within the inclusions. This MreB vs. PG degradation model also links MreB localization and movement to how, where, and when chlamydial PG will be made; directly by the incorporation of new PG material, and/or indirectly by down-regulating the activity of the degradation mechanism. While MreB co-localization with PG strands in enlarged aberrant bodies upon β-lactam inhibition of PBPs is consistent with this model, this observation also shows that transglycosylation in *Chlamydia* can be uncoupled from D,D-transpeptidation/carboxypeptidation. D,D-transpeptidation/carboxypeptidation may still play a role in defining the proper ring shape for division, but not for nascent PG assembly.

In conclusion, pathogenic Chlamydiae lack a classical PG sacculus and limit PG synthesis to a narrow PG ring during the replicative phase of their developmental cycle. We propose that the expression of minimal quantities of immunostimulatory PG represents pathoadaptive evolution by *Chlamydia*. Immune evasion by the restriction or modification of PAMPs is well-documented in other pathogenic microbes [63]. The reduced PG ring present within pathogenic *Chlamydia* species is likely the product of their adaptation to an obligate, intracellular lifestyle in which a full PG sacculus is not required and may even be detrimental to bacterial survival. We propose that this PG ring is made and re-sculpted to facilitate cell division and growth by the interplay of MreB with a PG degradation mechanism. This study also established a direct visual link between nascent bacterial PG synthesis and a functional and dynamic MreB. With the availability of inducible promoters[64, 65], gene inactivation systems [66] and allelic exchange [67] in *Chlamydia*, DAADs will allow future studies to explore the models proposed in this work by controlling various aspects of PG biosynthesis and degradation in intracellular, pathogenic *Chlamydia*. Our observation that inhibition of MreB has global inhibitory effects throughout the chlamydial life cycle not only makes *Chlamydia* an attractive organism in which to study MreB, but also presents this protein as a promising anti-chlamydial target for future studies.

## Materials and Methods

### Reagents

Clickable dipeptide PG probes (EDA—DA and ADA—DA) and MreB polymerization inhibitors (A22 and MP265) were synthesized as previously described[9, 49]. Alkynyl- and Azide-functionalized Alexa Fluor 488, TAMRA-5-azide, and Click-iT Cell Reaction Buffer Kit were purchased from Invitrogen. Antibodies against chlamydial MreB and IncA were generously provided by Scott Hefty (University of Kansas) and Dan Rocky (Oregon State University), respectively. The chlamydial RSGFP-expressing p2TK-SW2 plasmid was generously provided by Isabelle Derré (University of Virginia).

### Bacterial strains, growth conditions, and quantification of inclusion-forming units (IFUs)

*C*. *trachomatis* serovar L2 strain 434/Bu was provided by H. Caldwell (Rocky Mountain Laboratories). *C*. *muridarum* strains Nigg "M9"[68], *C*. *psittaci* strain 6BC "BCRB"[69], *C*. *caviae* strain GPIC "SP6" [70] are all clonal lab strains picked and expanded from single plaques. Chlamydial stocks were generated and asynchronous infections were performed as previously described[9]. Briefly, chlamydial EBs were harvested from infected L2 (mouse fibroblast) cells at 40 hpi and stored at -80°C until use. For infections, tissue culture-treated glass coverslips were placed in 24 well plates (Costar) and L2 cells were plated so as to reach ~70–80% confluence by the day infections were carried out. Cells were washed twice with warm Dulbecco's modified Eagle's medium (DMEM), infected at a multiplicity of infection (MOI) of 1 with bacteria resuspended in DMEM, plates were rocked for two hours at 37°C 5% CO_2_, unbound bacteria were subsequently removed, and medium was replaced with DMEM supplemented with 10% FBS (HyClone), 1 × MEM Non-Essential Amino Acids Solution (Sigma), and 0.2 μg ml^−1^ cycloheximide (Sigma). Medium was supplemented with native/modified dipeptide molecules, antibiotics, and/or MreB inhibitors as noted in the text. For quantification of the toxicity of MreB-polymerization inhibitors, infections were carried out as described above utilizing the *C*. *trachomatis* serovar L2 strain 434/Bu transformed with the p2TK-SW2 plasmid [71] for the expression of RSGFP. Inhibitors were added at the time points / durations indicated in the text. At either 44 or 52 hpi, 1 ml of sucrose/phosphate glutamate buffer (SPG) was added to each well and cells were collected via scraping with glass beads. Resuspended cells were then subjected to sonication for brief (ten second) pulses, 1:10 dilutions were prepared and were immediately used to infect 96 well monolayers of L2 cells, which were seeded the previous day with 200 μl of 200,000 L2 cells/ml per well. Plates were then spun in an Eppendorf desktop centrifuge at 3000 rpm 35C for 1 hour and then allowed to incubate at 37°C 5% CO_2_ for 24 hours. Inclusions were counted in live cells via fluorescence microscopy and IFUs were calculated for each experimental group in biological triplicates.

### Labeling of chlamydial PG

Experiments in which chlamydial PG was labeled with incorporated, clickable dipeptide probes (DAADs) were carried out as described previously[9]. Briefly, at designated time points post infection, treated coverslips containing *Chlamydia*-infected cells were washed first in warm DMEM, then with PBS, and fixed/ permeabilized with methanol for five minutes. Cells were then washed with PBS, further permeabilized with 0.5% Triton X for five minutes, and washed a final time with PBS. Coverslips were then blocked with 3% BSA (in PBS) and the click chemistry reaction was carried out utilizing the Click-iT Cell Reaction Buffer Kit (Invitrogen) with appropriate azide-modified fluorophores (Alexa fluor 488/647 and TAMRA-5). The addition of copper (cupric sulfate) results in the alkyne group on the dipeptide probe and the azide group on the fluorophore forming a stable triazole conjugate, thereby fluorescently labeling the PG in which the probe has been incorporated. Where indicated, chlamydial major outer membrane protein (MOMP) was labeled with anti-MOMP antibody (LifeSpan Biosciences, 1:500), chlamydial inclusions were labeled with anti-IncA antibody ([72], 1:500) and chlamydial MreB was labeled with anti-chlamydial MreB diluted 1:1,000. Alexa fluor-conjugated anti-mouse, anti-goat, and anti-rabbit secondary antibodies (Invitrogen) were diluted 1:2,000.

### Pulse chase experiments

L2 cells were infected with *C*. *trachomatis* serovar L2 strain 434/Bu for 16 hours. Infection medium was then removed, cells were washed once with pre-warmed DMEM, and new infection medium was added containing 4 mM EDA—DA (pulse). After one hour, medium was removed, cells were washed twice with pre-warmed DMEM, and new medium was added containing a second probe, small molecule inhibitor, or neither (chase). Cells were fixed, permeabilized at indicated time points, and labeling of chlamydial PG and MOMP was conducted, as described previously. For the pulse-chase experiment conducted in Fig 8, a similar principle was followed, but with the following differences. After a 2 h EDA—DA (4 mM) pulse, cells were washed and incubated in fresh medium containing MP265 (125 μM) for an additional 1 h (1^st^ Chase). At this point the cells were washed again and new medium (that lacked both EDA—DA or MP265) was added containing either antibiotics, D-Ala—D-Ala, or neither (2^nd^ Chase).

### Imaging and analysis

All confocal imaging was conducted with a Zeiss 710 laser scanning microscope utilizing Zen 2012 (Carl Zeiss) software and all epifluorescence imaging was conducted by a Nikon Ti-E inverted fluorescence microscope equipped with a Plan Apo 60x/1.40 Oil Ph3 DM objective and a DAPI/GFP/Cy3/Cy5 filter cube and an Andor DU885 EMCCD camera. Settings were fixed at the beginning of image acquisition and for experiments in which different samples/time points were to be compared, the same parameters were applied for collecting and post processing all images taken. Deconvolution and maximum intensity projection (when used) was conducted utilizing AxioVision (Carl Zeiss) software employing the inverse filter setting or ImageJ, respectively. ImageJ was used for all subsequent image analysis. For generating the data presented for the kinetic analysis of the average fluorescence of chlamydial inclusions, confocal Z-stacks were taken using the 40x objective and maximum intensity projections were generated from those stacks and pixels assigned a brightness level between 0 and 255. Basic intensity quantification was conducted by using the MOMP-labeling (red) channel to define the area which encompassed individual inclusions, and this was then used as an overlay to subsequently quantify the level of fluorescence present within each inclusion area present within the EDA—DA (green) channel.

3D SIM super-resolution microscopy was performed on a Delta Vision OMX microscope equipped with an Olympus 100X/1.40 Oil PSF objective and a Photometrics Cascade II EMCCD camera. The samples were excited with lasers at 405 nm, 488 nm, 561 nm, 642 nm and the emission was detected through 419 nm -465 nm, 500 nm -550 nm, 609 nm -654 nm, 665 nm -705 nm emission filters. The image processing was conducted by SoftWorx imaging software. Further image analysis was conducted via ImageJ including the measurements of PG ring widths. For PG width measurements, rings that were aligned perpendicular to the x,y axis (for the maximum resolution) were used. SIM imaging Z stacks of chlamydial inclusions were obtained via a Zeiss ELYRA PS.1 utilizing Zen 2012 (Carl Zeiss) software for image processing.

### Chlamydial muropeptide isolation

Muropeptide fragments of PG were isolated from *Chlamydia*-infected cells as previously described [50]. Two 175 cm^2^ flasks of confluent HeLa cells were either infected with *C*. *trachomatis* L2/434 at an MOI of 1 or mock infected and incubated for two hours at 37°C in 5% CO_2_ with rocking. Infection medium was then removed and replaced with DMEM with heat inactivated fetal bovine serum (10%), and cells incubated for an additional 16 hours. Medium was then removed and replaced with fresh DMEM (10% FBS) with or without 75 μM compound A22 and cells were then allowed to incubate an additional two hours. Medium was removed, cells were washed once with warm DMEM (to remove any residual A22) and then cells were harvested using glass beads, resuspended in DMEM, and sonicated at 40 amps with one-minute pulses, repeated five times. Supernatants were centrifuged at 4,000 g for five minutes to remove cellular debris, lysates were passed through a 0.22 μm filter, and then 3 kDa centrifugal filters (Amicon UFC900324) at 4000 x g for one hour at 37°C. Flow-through fractions from the 3 kDa centrifugal filters were then heat inactivated at 95°C for six minutes and assayed for activity in an HEK NOD reporter cell line (see below).

### NOD1 / NOD2 NF-κB reporter assay

The NOD signaling assay was conducted on lysates of *Chlamydia*-infected cells as previously described. Briefly, HEK cells overexpressing either the NOD1 or NOD2 receptors, as well as the NF-κB-SEAP reporter gene (Invivogen, CA), were used to quantify the immunostimulatory potential from cell lysate fractions taken from a reverse phase C18 HPLC column. Twenty μl of cell lysate fractions from mock-infected and *Chlamydia*-infected cells (in the presence or absence of compound A22) were added to ~5 x 10^4^ HEK-Blue NOD1 or NOD2 cells in 96 well plates (total reaction volume 200 μl/well) and incubated for 24 hrs at 37°C. Secreted alkaline phosphatase (SEAP) was measured by adding 20 μl of the supernatant from lysate fraction-treated wells to 180 μl of QUANTI-blue substrate (Invivogen) in a separate 96 well microtiter plate. Untreated or uninfected cell supernatants were used as negative controls. The reaction was incubated at 37°C for 30 min and SEAP activity was assessed by reading OD at 650 nm.

### PG analysis

HPLC was carried out on filtered, *Chlamydia*-infected cell lysates (and controls) as previously described [50]. LCMS experiments were performed on an Agilent 1200 Series liquid chromatography system coupled to an AB Sciex Q-Trap 4000 mass spectrometer with a Turbo V electrospray ionization source, as previously described [50]. Data from control/experimental groups was analyzed and overlaid using Analyst Software (v1.5.1) with all experiments and subsequent analysis conducted at least twice.

## Supporting Information

S1 Fig Analysis of chlamydial inclusion fluorescence. Epifluorescence (EPI) images of exponential *Staphylococcus aureus* cells incubated for 2 h with 0.5 mM EDA—DA (shown as a control for labeling for PG-labeled *Chlamydia* in Figs 2 and 3). Scale bar = 1 μm.
(TIF)

S2 Fig Single, 2D Z stacks are superior to 3D SIM projections in establishing individual cell boundaries of *Chlamydia* RBs closely packed within inclusions. Visualization of PG (green) and MOMP (red) for a chlamydial inclusion 18 hpi, as viewed by either individual 2D Z stacks or the rendered 3D projection. All stacks are visualized in S1 Video.
(TIF)

S3 Fig Individual imaging planes from SIM Z stacks allow for visualization of defined cell boundaries and confirms chlamydial PG localized to the division septum in actively dividing RBs. Single imaging planes of *C*. *trachomatis* infected cells incubated with 4 mM EDA-DA at 2 hpi and fixed at 18 hpi. Arrows indicate areas of punctate PG staining or polar disk formation. Instances of multiple or asymmetric PG ring localization are marked with arrowheads. MOMP and PG labeling is the same as in Fig 2. Images are representative of 30 inclusions analyzed. Scale bar = 1 μm. Panels **d** and **f** are separate imaging planes of the same inclusions. All stacks for panels **d, f,** and **e** and are visualized in S2 and S3 Videos.
(TIF)

S4 Fig Analysis of chlamydial inclusion fluorescence. **(a)** Data from a quantitative analysis of average fluorescence pixel intensities (presented in Fig 5b and 5c) were re-plotted against inclusion size (area) for both PG and MOMP labeling channels. **(b)** Inclusion area (as measured by MOMP-labeled surface area) is graphed for all groups presented in Fig 5b and 5c. **(c)** Comparison of the average inclusion pixel fluorescence intensity values from an untreated control group (no EDA—DA added) and inclusions grown in medium containing 4 mM EDA—DA for either one or five hours. Error bars represent standard deviation of the mean.
(TIF)

S5 Fig Effects of polymerization inhibitors on MreB localization. **(a)** Chlamydial EBs were allowed to differentiate into RBs, mature for 18 hours in the presence or absence of D-cycloserine and then subjected to treatment with MreB polymerization inhibitor A22 for one hour. Scale bars = 1 and 3 μm, respectively. **(b)** SIM of MreB and PG labeling (EDA-DA) for EBs (arrowhead) and RBs (larger cells on the left) at 18 hpi. Scale bar = 1 μm.
(TIF)

S6 Fig Short pulse EDA-DA labeling results in patchy PG localization that partially co-localizes to MreB patches. **(a)** Maximum intensity projection of EDA—DA signal within chlamydial inclusions (18 hpi) incubated five min with 4 mM EDA—DA. A RB with only a partially labeled PG ring is denoted by red arrowhead. Scale bar = 1 μm. **(b)** SIM of EDA—DA labeled PG (green) and MreB (red) within chlamydial inclusions (18 hpi) incubated with 4 mM EDA—DA for 5 minutes. Yellow arrowheads indicate areas of co-localization and white arrowheads denote the localization of MreB patches complementary to newly-forming PG arcs. Scale bars = 1 μm.
(TIF)

S7 Fig Mass spec analysis of the effects of MreB inhibition on chlamydial PG muropeptide abundance. (**a**) NOD2 signaling analysis of *Chlamydia*-infected cell lysates grown in the presence/absence of MreB inhibitor A22 (75 μM) for two hours. Signaling assays were conducted in quadruplicate and results are representative of two independent experiments. Error bars represent standard deviation of the mean. **(b)** Comparison of extracted ion current (XIC) of 477.2 *m/z* (muramyl dipeptide fragment at Rt of 8.4 min) between infected, untreated (red line) and infected, A22-treated (blue line) NOD2-activating fractions. Image is representative of three separate analyses conducted on three separate biological replicates. The increase in the intensity of ion 477.2 *m/z* observed in A22-treated cell lysates over untreated infected lysates was quantitated by calculating the area under the respective peaks and is shown in the table (c). **(c)** The intensities of muropeptide ions 477.2m/z, 653.2 m/z, 494.2 *m/z* and 666.2 *m/z* (Rt 8.4, 9.4, 7.5 and 8.1 min respectively) were quantitated from the XIC of infected A22-treated and untreated lysates and the data are presented in the upper half of the table. The effects of DCS treatment on the intensity of the same four muropeptide ions are presented in the lower half of the table. **(d)** Breakdown products produced from the 477.2 m/z ion when subjected to MS/MS. The resulting spectra correspond to the known (partial) structure of muramyl dipeptide (*MDP)* from *C*. *trachomatis* [50].
(TIF)

S8 Fig Chlamydial MreB is essential for the EB-RB transition, inclusion maturation and growth. **(a)** Inclusion sizes (at 24 hpi) as measured by MOMP-labeling subsequent to the addition of A22 at various time points post-infection. **(b)** Quantitative analysis of the distribution of size of all intracellular *Chlamydia* distinguished by MOMP labeling 24 hpi comparing cells either untreated or treated with MreB-inhibitors MP265 or A22 at 2 hpi, which were then either left on or removed 10 hpi. **(c)** Maximum intensity projection of MOMP (red) and DAPI (blue) of chlamydial inclusions (18 hpi) grown in the presence of A22 (added 2 hpi). **(d)** Recovered inclusion forming units (IFUs) representing viable EBs collected (at either 44 hpi or 52 hpi, as indicated) after treatment with MreB polymerization inhibitors. Inhibitors were added (and removed) at specific time points throughout the chlamydial developmental cycle for the indicated durations. Each toxicity assay was conducted in triplicate, error bars represent standard deviation of the mean, and data are representative of two independent biological replicates. UTD; untreated control. **(e)** Chlamydial inclusions (24 hpi) allowed to develop in the presence/absence of A22 or DCS and labeled with anti-_CT_IncA antibody (green), anti-_CT_MOMP (red), and DAPI (blue). All images are maximum intensity projections of confocal Z-stacks, and are representative of over 30 inclusions viewed by confocal microscopy (and >100 viewed by epifluorescence microscopy). Each study spanned two independent experiments. **(f)** Chlamydial inclusions 22 hpi. Cells were either left untreated (upper panel) or treated with the MreB inhibitor A22 at 2 hpi, which was then either left in (middle) or removed 10 hpi (lower panel). Labels are described in panel captions. Scale bar = 1 μm.
(TIF)

S9 Fig Effects of β-lactams and D-cycloserine on chlamydial MreB and PG. Maximum intensity projections of chlamydial inclusions that have been incubated with ampicillin **(a)** or piperacillin **(b)** and labeled with EDA-DA for 1 hour. **(c)** EPI maximum intensity projections of ampicillin-induced aberrant bodies pulsed with EDA—DA for one hour prior to fixation and staining for EDA—DA (upper panel) and after additional 2 h treatment with 200 μg lysozyme ml^−1^ (lower panel). **(d)** Maximum intensity projections of chlamydial aberrant bodies (induced by adding ampicillin to the growth medium at 2 hpi) grown in the presence of 4 mM EDA—DA for one hour prior to fixation and staining for MreB. **(e)** Chlamydial aberrant bodies (induced by adding DCS to the medium at 2 hpi) labeled for MreB. Labels are described in panel captions. **(e)** Scale bars: **a** = 5 μm. **b-f** = 1 μm.
(TIF)

S10 Fig Increase in inclusion size does not account for changes in PG labeling intensity. **(a)** Schematic representation of the homogenous vs. bidirectional removal of the older PG. In contrast to the homogeneous degradation of PG about the entire ring, the bidirectional model predicts splitting of the older ring (green) into two with new PG (pink) being inserted in the middle. **(b-c)** Inclusion area (as measured by MOMP labeling) graphed for all groups and PG (black) / MOMP (red) labeling re-plotted against inclusion size. Inclusion size was measured to ensure that changes in fluorescence intensity were not simply attributable to inclusion growth over time. The total inclusion size trended upward three hours after the initial DAAD pulse (two hours after the beginning of the chase portion of the experiment (**b**)), however, this did not appear to affect average fluorescence intensities within any given experimental group (**c**).
(TIF)

S11 Fig Limitations of clickable dipeptide probes for use in kinetic studies. **(a)** Pulse chase experiments in *Chlamydia* with competitive, native dipeptide (DA—DA) supplemented into fresh medium and added to clickable, EDA—DA labeled inclusions. The inclusions that are sequentially pulsed with EDA—DA and DA-DA in order to show the effect that lengthy exposures to native DA-DA pools has on the loss of EDA—DA signal over time. **(b)** The same pulse chase experiment presented in panel **a**, but carried out over one hour and comparing the effects of exogenously added ADA-DA on loss of EDA-DA fluorescence. The addition of ADA—DA results in a drastic decrease in EDA—DA signal, most likely because incorporated azide containing ADA—DA outcompetes the Alexa Fluor 488 azide during the click chemistry reaction, i.e. most of the EDA—DA labeled PG is captured by ADA—DA labeled PG instead of the Alexa Fluor 488 that is used for the read-out of the EDA—DA labeled PG. For both experiments, average fluorescence values were calculated for each treatment group at the indicated time points, subsequent to chases. Each bar represents the average fluorescent pixel intensities of ~150 chlamydial inclusions pooled from two independent experiments. Error bars represent the standard deviation of the mean for each sample group. *Chlamydia* PG and MOMP were labeled as described in Fig 2.
(TIF)

S1 Video SIM Z-stacks (presented as single imaging planes in S2 Fig) of *Chlamydia* inclusions 18 hpi taken via Zeiss ELYRA PS.1. PG is labeled in green and MOMP in red. Movies begin with 3D-rendered projections of each Z stack rotated about the X or Y axis followed immediately by the presentation of each individual imaging plane successively through the Z stack of the inclusion. Single channels are presented first and followed by a merged compilation of the two.
(WMV)

S2 Video SIM Z-stacks (presented as single imaging planes in S3d and S3f Fig) of *Chlamydia* inclusions 18 hpi taken via Zeiss ELYRA PS.1. PG is labeled in green and MOMP in red. Movies begin with 3D-rendered projections of each Z stack rotated about the X or Y axis followed immediately by the presentation of each individual imaging plane successively through the Z stack of the inclusion. Single channels are presented first and followed by a merged compilation of the two.
(WMV)

S3 Video SIM Z-stacks (presented as single imaging planes in S3e Fig) of *Chlamydia* inclusions 18 hpi taken via Zeiss ELYRA PS.1. PG is labeled in green and MOMP in red. Individual imaging planes are presented successively through the Z stack of the inclusion. Single channels are presented first and followed by a merged compilation of the two.
(WMV)

S4 Video SIM Z-stacks of *Chlamydia* inclusions 18 hpi taken via Zeiss ELYRA PS.1. PG is labeled in green and MOMP in red. Movies begin with 3D-rendered projections of each Z stack rotated about the X or Y axis followed immediately by the presentation of each individual imaging plane successively through the Z stack of the inclusion. Single channels are presented first and followed by a merged compilation of the two.
(WMV)
