# Supplementary figures and images for: Pathogenic Chlamydia Lack a Classical Sacculus but Synthesize a Narrow, Mid-cell Peptidoglycan Ring, Regulated by MreB, for Cell Division
Source: PLoS Pathog. 2016 May 4;12(5):e1005590. doi: 10.1371/journal.ppat.1005590 (PMC4856321; doi:10.1371/journal.ppat.1005590)

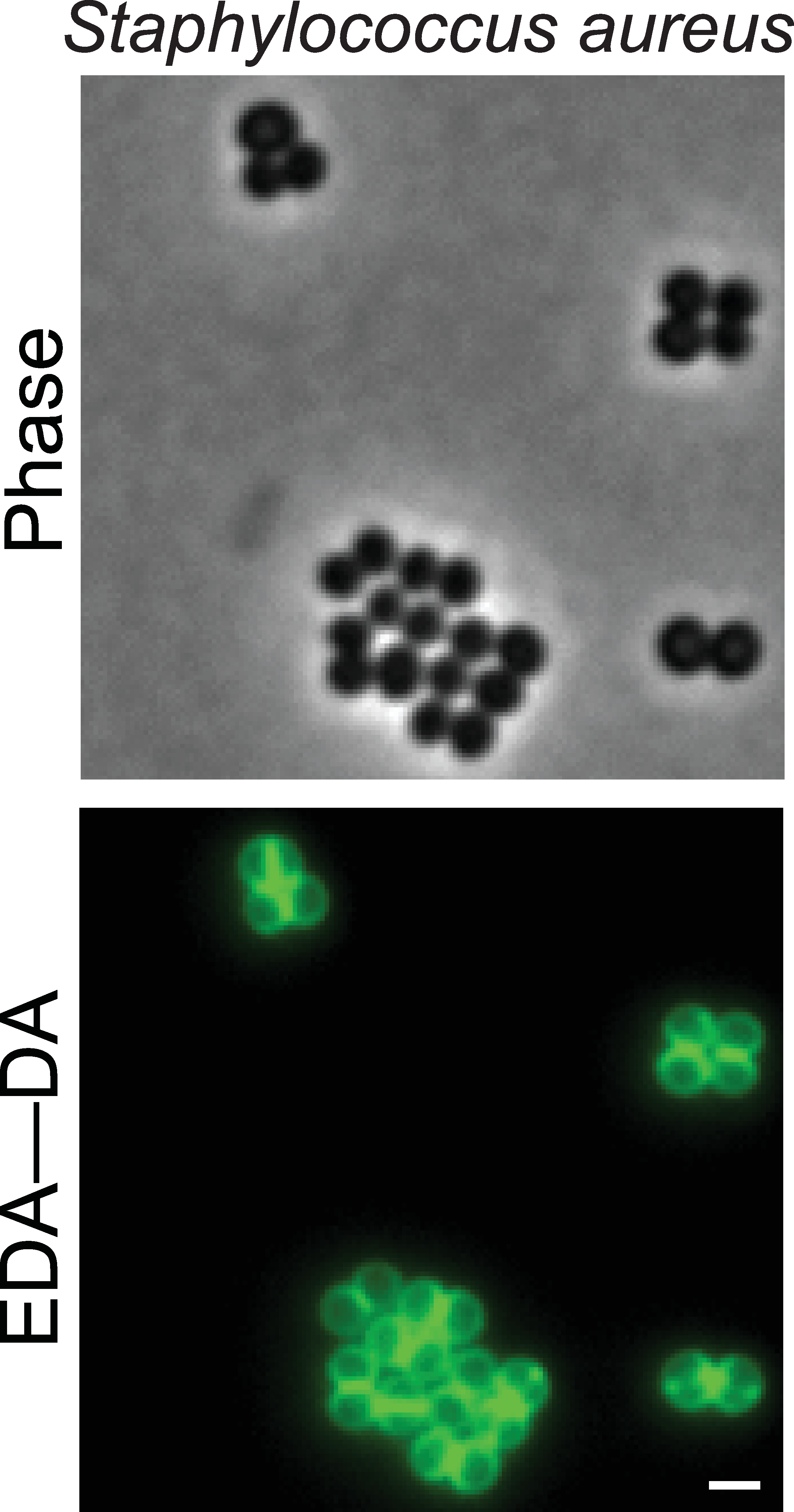

Supplement: S1 Fig — Epifluorescence (EPI) images of exponential Staphylococcus aureus cells incubated for 2 h with 0.5 mM EDA—DA (shown as a control for labeling for PG-labeled Chlamydia in Figs 2 and 3). Scale bar = 1 μm. (TIF) [file ppat.1005590.s001.tif]

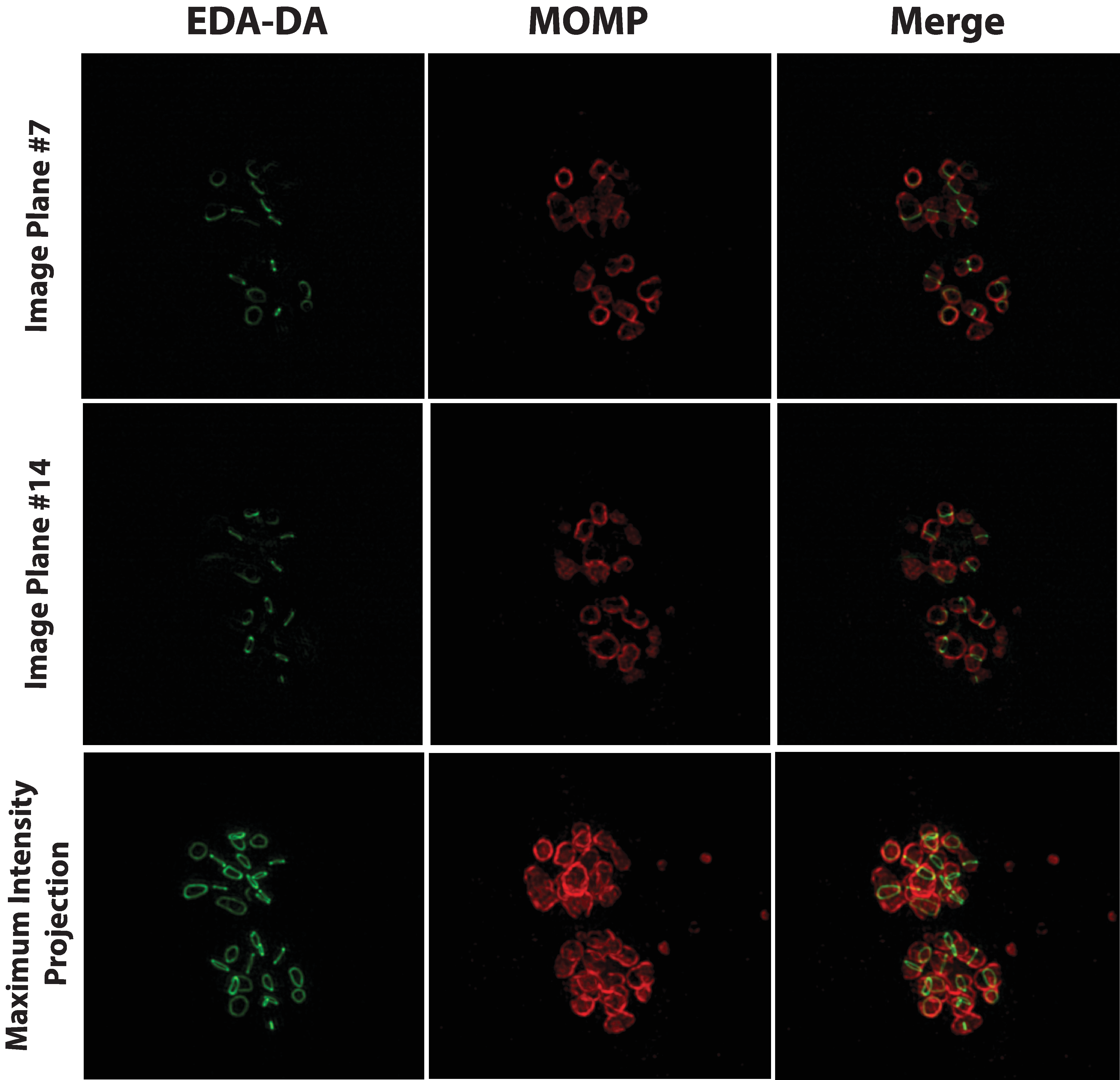

Supplement: S2 Fig — Visualization of PG (green) and MOMP (red) for a chlamydial inclusion 18 hpi, as viewed by either individual 2D Z stacks or the rendered 3D projection. All stacks are visualized in S1 Video. (TIF) [file ppat.1005590.s002.tif]

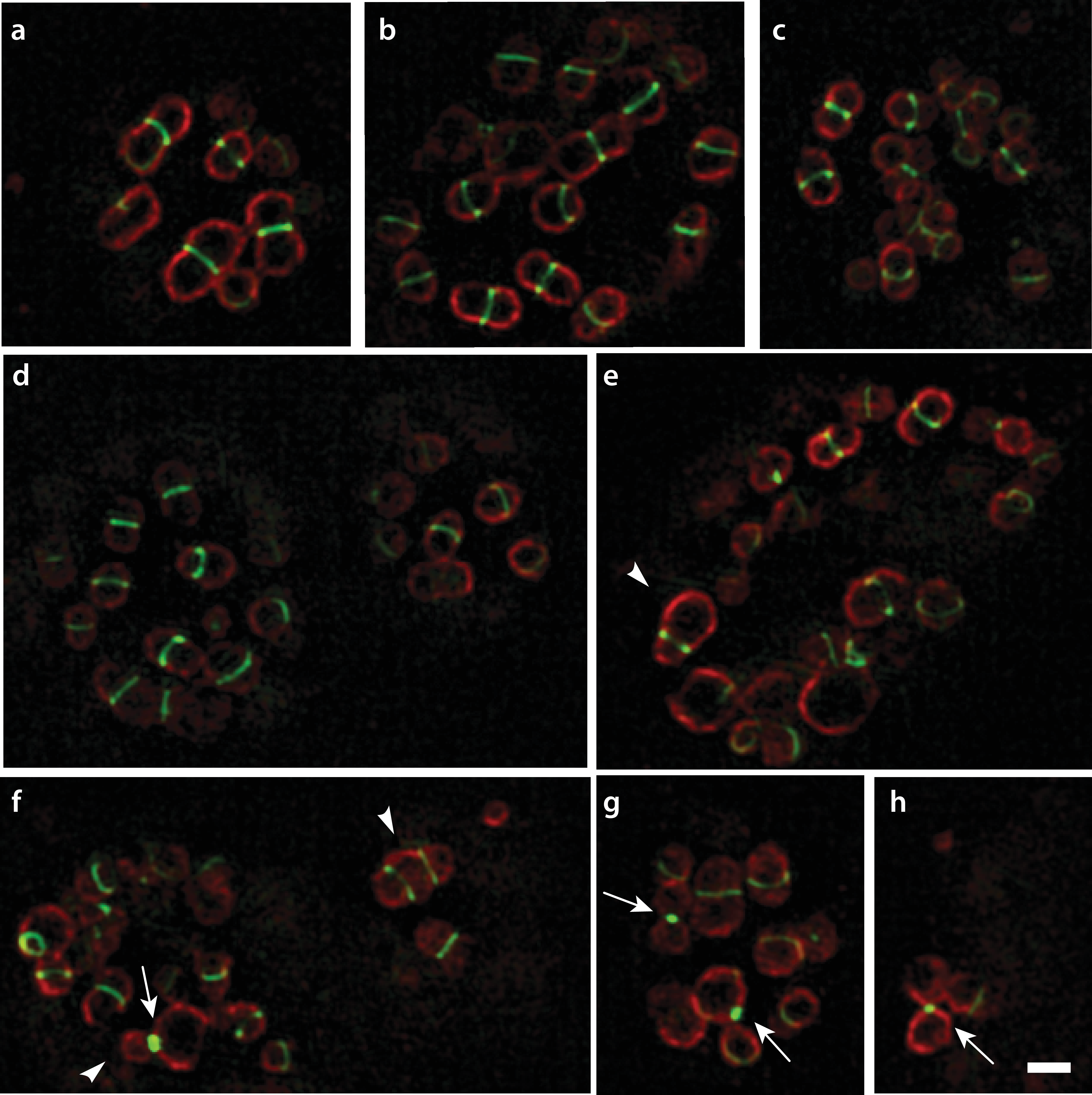

Supplement: S3 Fig — Single imaging planes of C. trachomatis infected cells incubated with 4 mM EDA-DA at 2 hpi and fixed at 18 hpi. Arrows indicate areas of punctate PG staining or polar disk formation. Instances of multiple or asymmetric PG ring localization are marked with arrowheads. MOMP and PG labeling is the same as in Fig 2. Images are representative of 30 inclusions analyzed. Scale bar = 1 μm. Panels d and f are separate imaging planes of the same inclusions. All stacks for panels d, f, and e and are visualized in S2 and S3 Videos. (TIF) [file ppat.1005590.s003.tif]

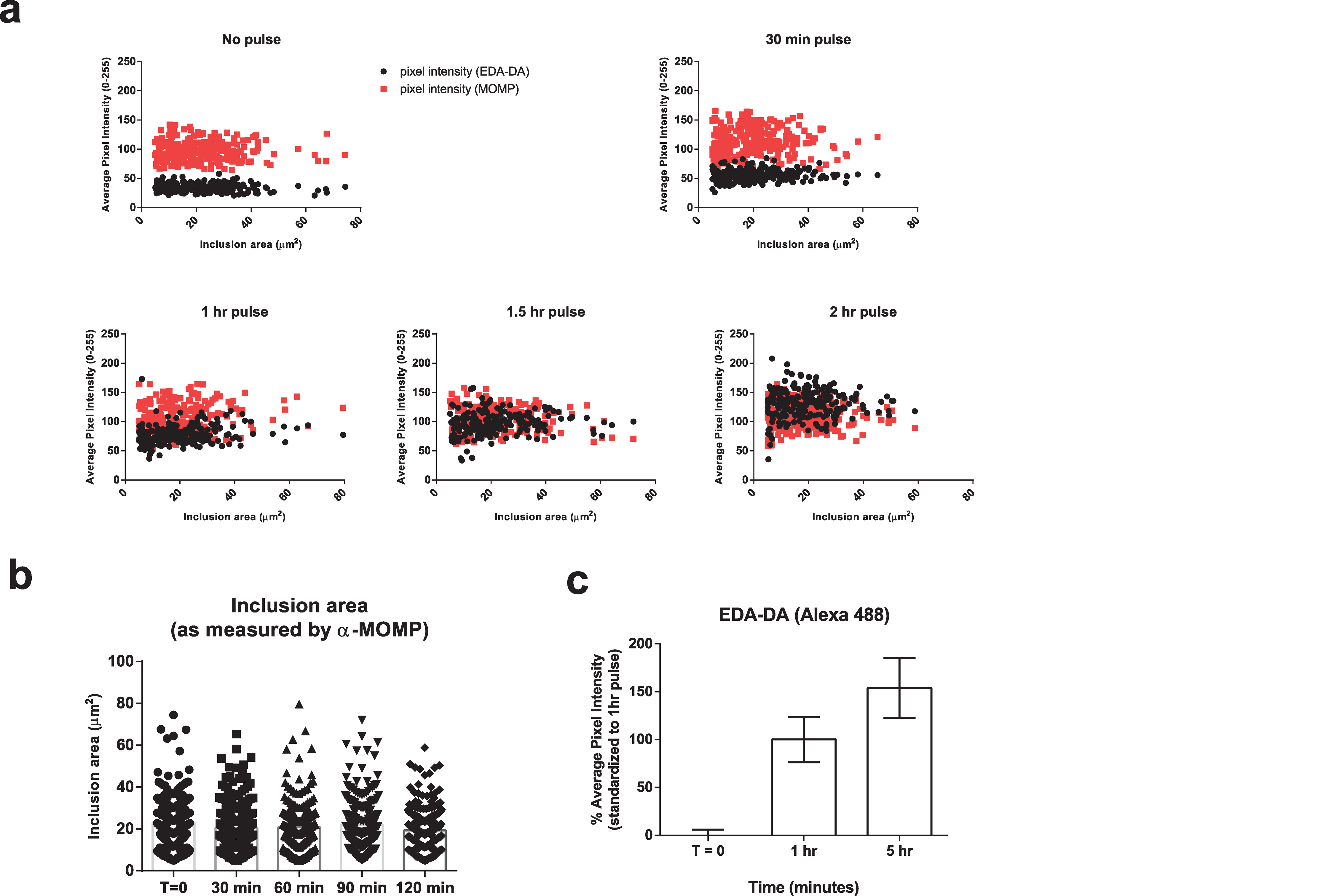

Supplement: S4 Fig — (a) Data from a quantitative analysis of average fluorescence pixel intensities (presented in Fig 5b and 5c) were re-plotted against inclusion size (area) for both PG and MOMP labeling channels. (b) Inclusion area (as measured by MOMP-labeled surface area) is graphed for all groups presented in Fig 5b and 5c. (c) Comparison of the average inclusion pixel fluorescence intensity values from an untreated control group (no EDA—DA added) and inclusions grown in medium containing 4 mM EDA—DA for either one or five hours. Error bars represent standard deviation of the mean. (TIF) [file ppat.1005590.s004.tif]

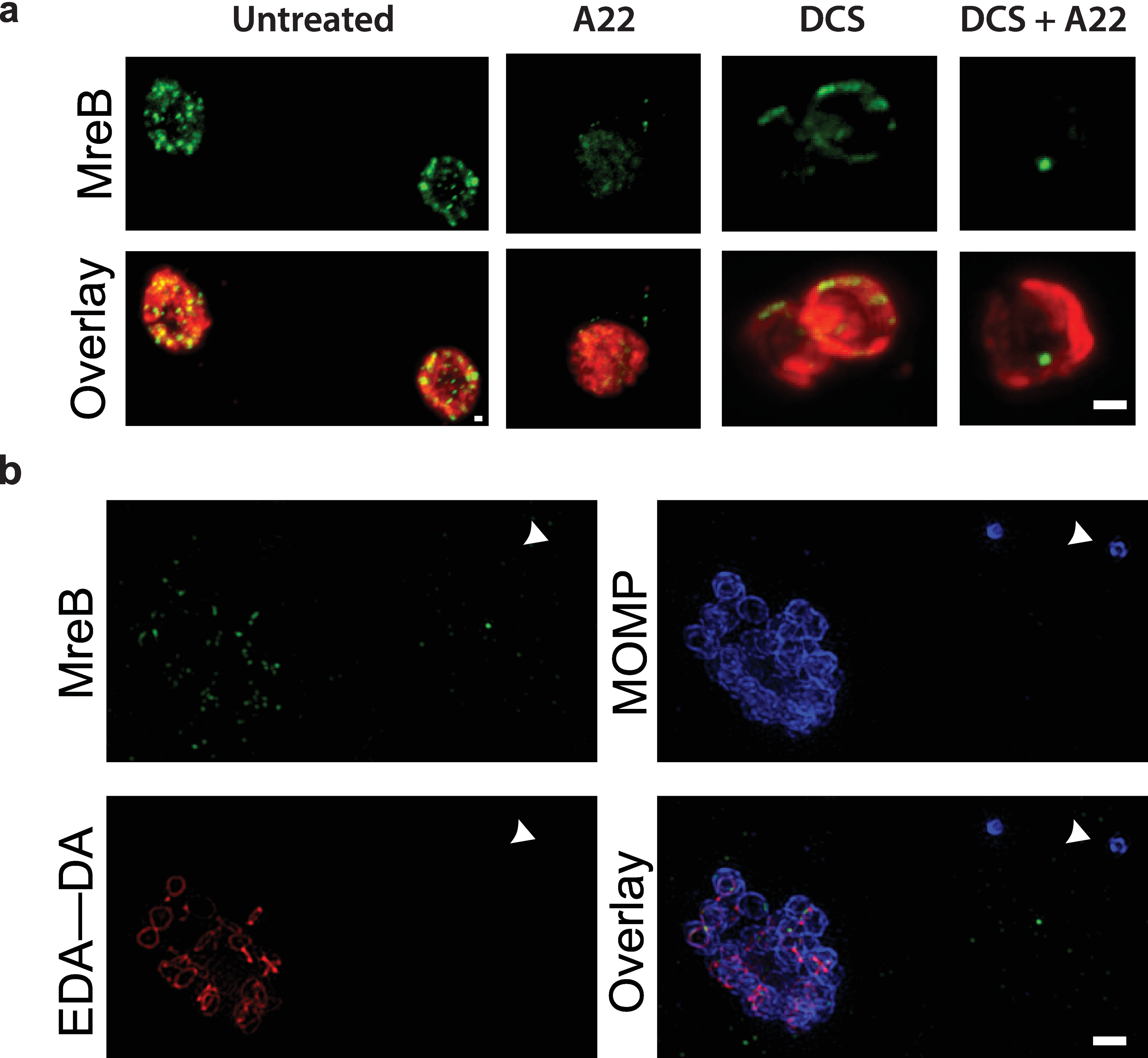

Supplement: S5 Fig — (a) Chlamydial EBs were allowed to differentiate into RBs, mature for 18 hours in the presence or absence of D-cycloserine and then subjected to treatment with MreB polymerization inhibitor A22 for one hour. Scale bars = 1 and 3 μm, respectively. (b) SIM of MreB and PG labeling (EDA-DA) for EBs (arrowhead) and RBs (larger cells on the left) at 18 hpi. Scale bar = 1 μm. (TIF) [file ppat.1005590.s005.tif]

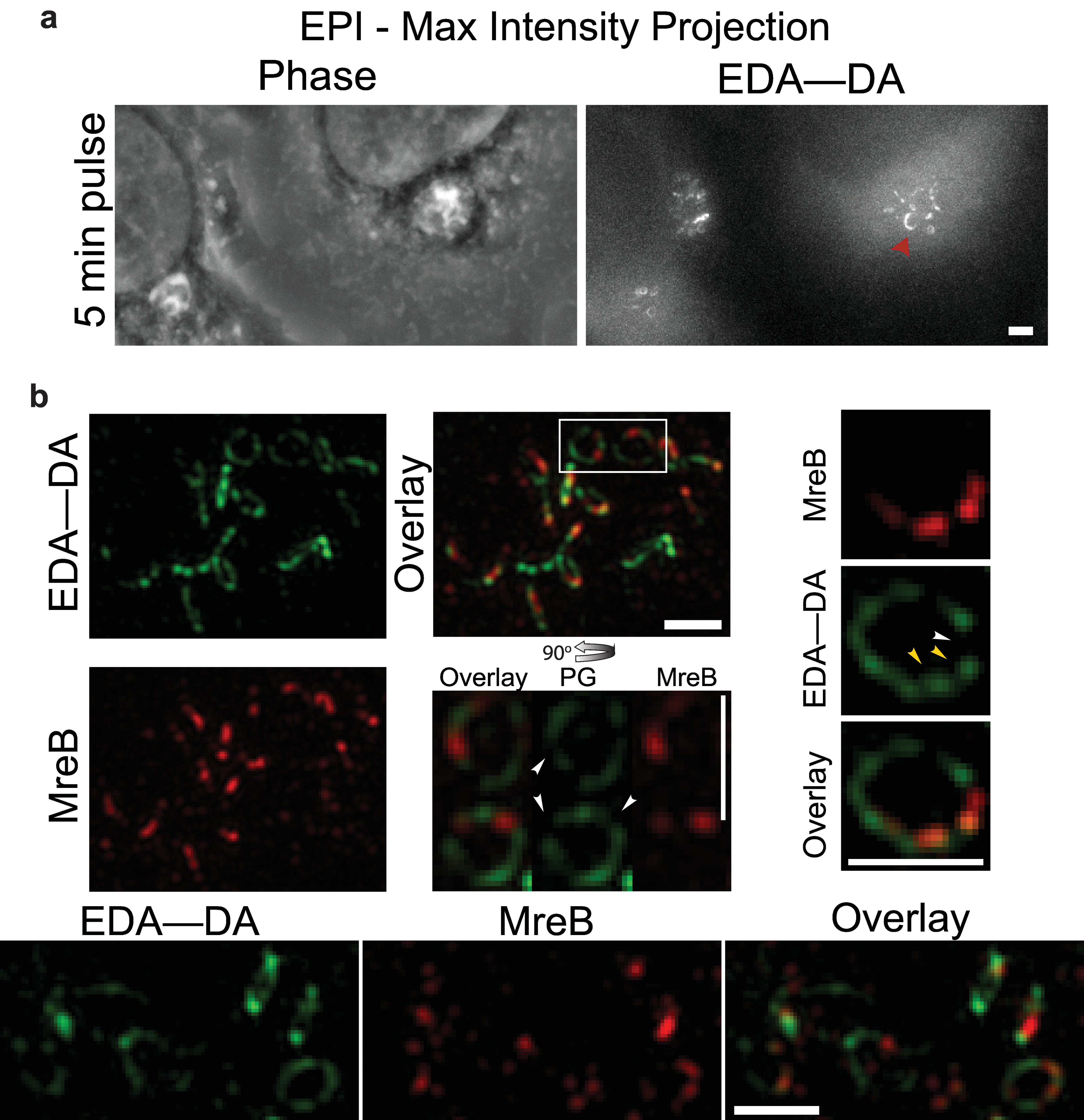

Supplement: S6 Fig — (a) Maximum intensity projection of EDA—DA signal within chlamydial inclusions (18 hpi) incubated five min with 4 mM EDA—DA. A RB with only a partially labeled PG ring is denoted by red arrowhead. Scale bar = 1 μm. (b) SIM of EDA—DA labeled PG (green) and MreB (red) within chlamydial inclusions (18 hpi) incubated with 4 mM EDA—DA for 5 minutes. Yellow arrowheads indicate areas of co-localization and white arrowheads denote the localization of MreB patches complementary to newly-forming PG arcs. Scale bars = 1 μm. (TIF) [file ppat.1005590.s006.tif]

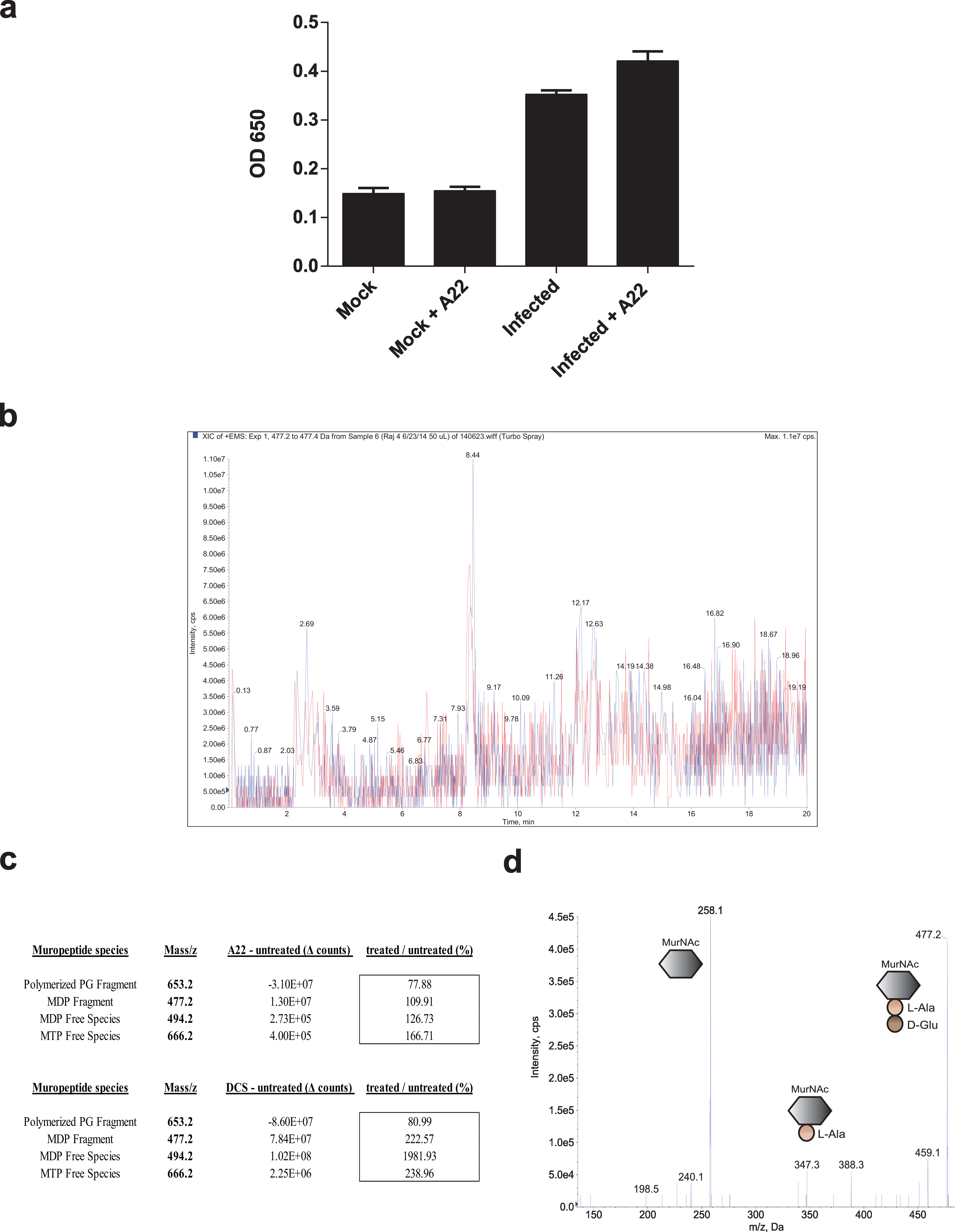

Supplement: S7 Fig — (a) NOD2 signaling analysis of Chlamydia-infected cell lysates grown in the presence/absence of MreB inhibitor A22 (75 μM) for two hours. Signaling assays were conducted in quadruplicate and results are representative of two independent experiments. Error bars represent standard deviation of the mean. (b) Comparison of extracted ion current (XIC) of 477.2 m/z (muramyl dipeptide fragment at Rt of 8.4 min) between infected, untreated (red line) and infected, A22-treated (blue line) NOD2-activating fractions. Image is representative of three separate analyses conducted on three separate biological replicates. The increase in the intensity of ion 477.2 m/z observed in A22-treated cell lysates over untreated infected lysates was quantitated by calculating the area under the respective peaks and is shown in the table (c). (c) The intensities of muropeptide ions 477.2m/z, 653.2 m/z, 494.2 m/z and 666.2 m/z (Rt 8.4, 9.4, 7.5 and 8.1 min respectively) were quantitated from the XIC of infected A22-treated and untreated lysates and the data are presented in the upper half of the table. The effects of DCS treatment on the intensity of the same four muropeptide ions are presented in the lower half of the table. (d) Breakdown products produced from the 477.2 m/z ion when subjected to MS/MS. The resulting spectra correspond to the known (partial) structure of muramyl dipeptide (MDP) from C. trachomatis [50]. (TIF) [file ppat.1005590.s007.tif]

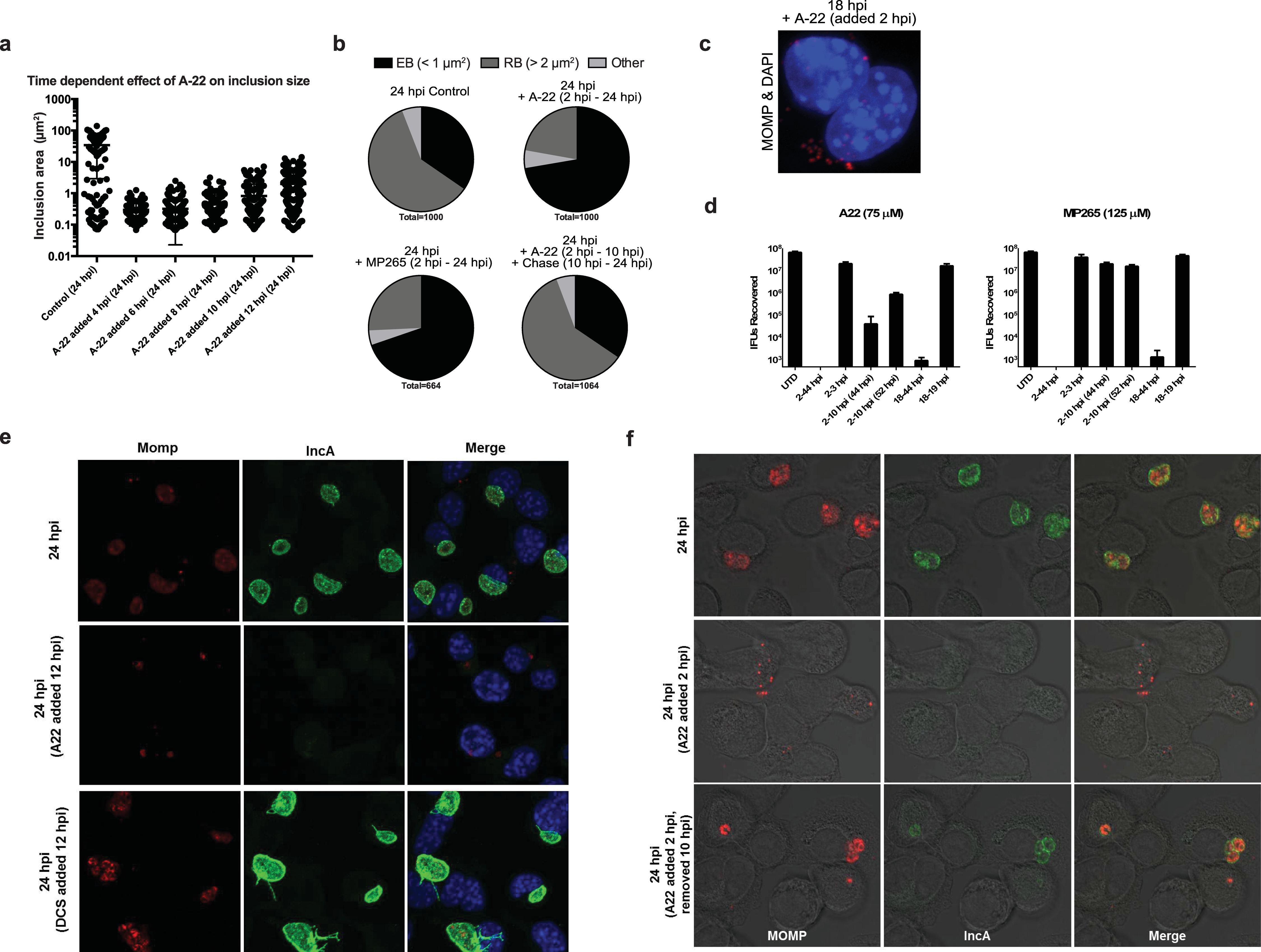

Supplement: S8 Fig — (a) Inclusion sizes (at 24 hpi) as measured by MOMP-labeling subsequent to the addition of A22 at various time points post-infection. (b) Quantitative analysis of the distribution of size of all intracellular Chlamydia distinguished by MOMP labeling 24 hpi comparing cells either untreated or treated with MreB-inhibitors MP265 or A22 at 2 hpi, which were then either left on or removed 10 hpi. (c) Maximum intensity projection of MOMP (red) and DAPI (blue) of chlamydial inclusions (18 hpi) grown in the presence of A22 (added 2 hpi). (d) Recovered inclusion forming units (IFUs) representing viable EBs collected (at either 44 hpi or 52 hpi, as indicated) after treatment with MreB polymerization inhibitors. Inhibitors were added (and removed) at specific time points throughout the chlamydial developmental cycle for the indicated durations. Each toxicity assay was conducted in triplicate, error bars represent standard deviation of the mean, and data are representative of two independent biological replicates. UTD; untreated control. (e) Chlamydial inclusions (24 hpi) allowed to develop in the presence/absence of A22 or DCS and labeled with anti-CTIncA antibody (green), anti-CTMOMP (red), and DAPI (blue). All images are maximum intensity projections of confocal Z-stacks, and are representative of over 30 inclusions viewed by confocal microscopy (and >100 viewed by epifluorescence microscopy). Each study spanned two independent experiments. (f) Chlamydial inclusions 22 hpi. Cells were either left untreated (upper panel) or treated with the MreB inhibitor A22 at 2 hpi, which was then either left in (middle) or removed 10 hpi (lower panel). Labels are described in panel captions. Scale bar = 1 μm. (TIF) [file ppat.1005590.s008.tif]

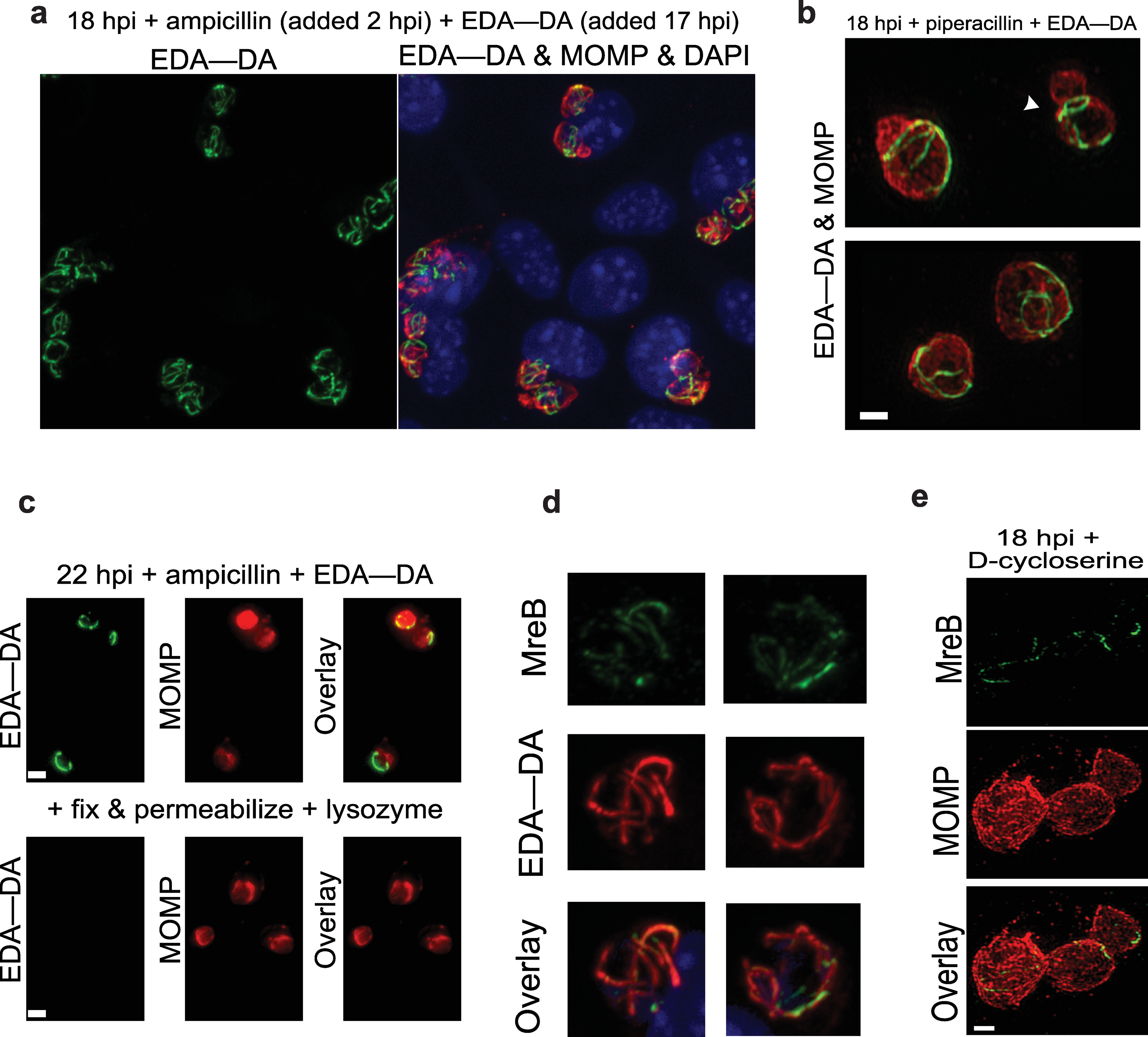

Supplement: S9 Fig — Maximum intensity projections of chlamydial inclusions that have been incubated with ampicillin (a) or piperacillin (b) and labeled with EDA-DA for 1 hour. (c) EPI maximum intensity projections of ampicillin-induced aberrant bodies pulsed with EDA—DA for one hour prior to fixation and staining for EDA—DA (upper panel) and after additional 2 h treatment with 200 μg lysozyme ml−1 (lower panel). (d) Maximum intensity projections of chlamydial aberrant bodies (induced by adding ampicillin to the growth medium at 2 hpi) grown in the presence of 4 mM EDA—DA for one hour prior to fixation and staining for MreB. (e) Chlamydial aberrant bodies (induced by adding DCS to the medium at 2 hpi) labeled for MreB. Labels are described in panel captions. (e) Scale bars: a = 5 μm. b-f = 1 μm. (TIF) [file ppat.1005590.s009.tif]

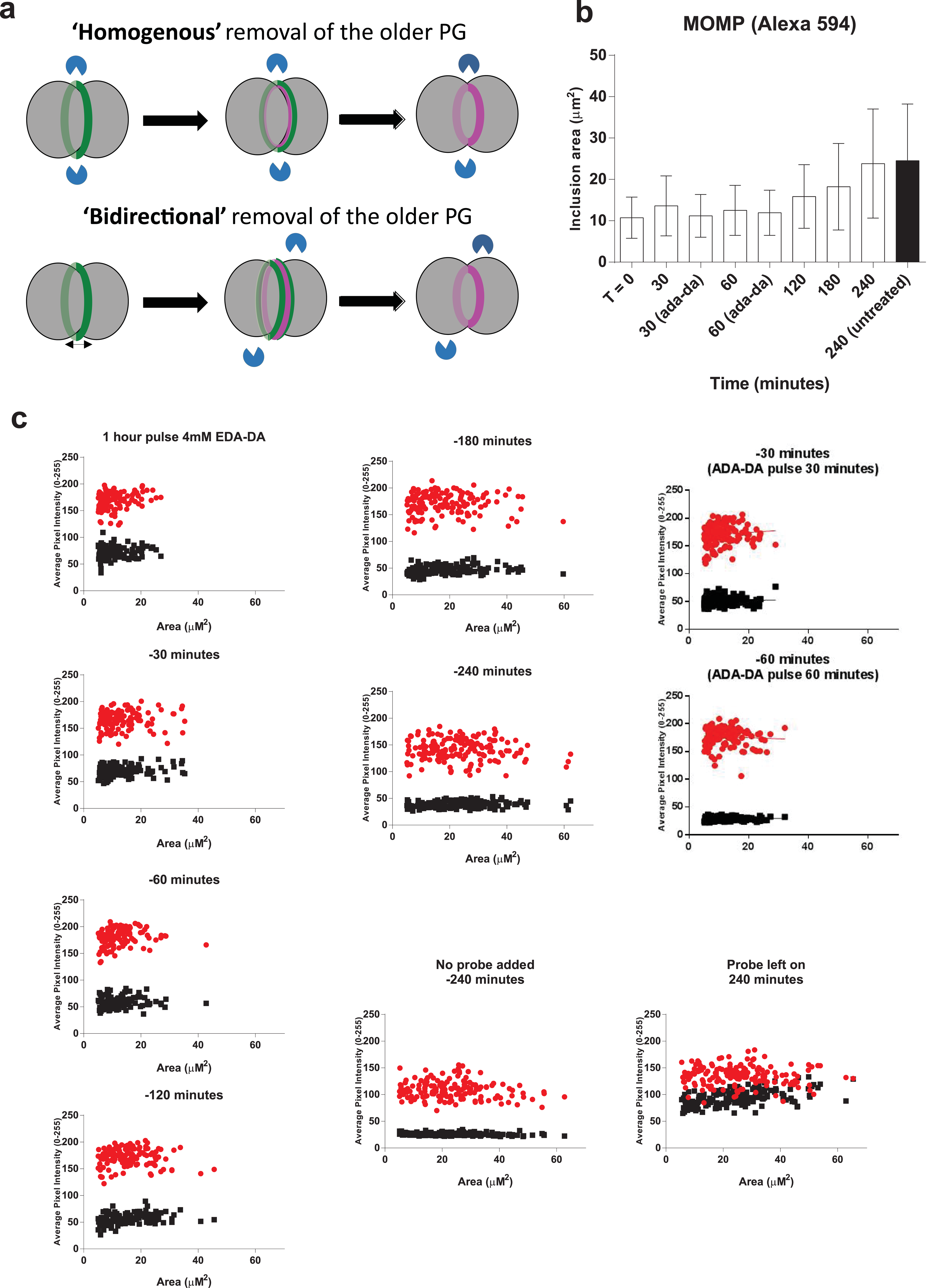

Supplement: S10 Fig — (a) Schematic representation of the homogenous vs. bidirectional removal of the older PG. In contrast to the homogeneous degradation of PG about the entire ring, the bidirectional model predicts splitting of the older ring (green) into two with new PG (pink) being inserted in the middle. (b-c) Inclusion area (as measured by MOMP labeling) graphed for all groups and PG (black) / MOMP (red) labeling re-plotted against inclusion size. Inclusion size was measured to ensure that changes in fluorescence intensity were not simply attributable to inclusion growth over time. The total inclusion size trended upward three hours after the initial DAAD pulse (two hours after the beginning of the chase portion of the experiment (b)), however, this did not appear to affect average fluorescence intensities within any given experimental group (c). (TIF) [file ppat.1005590.s010.tif]

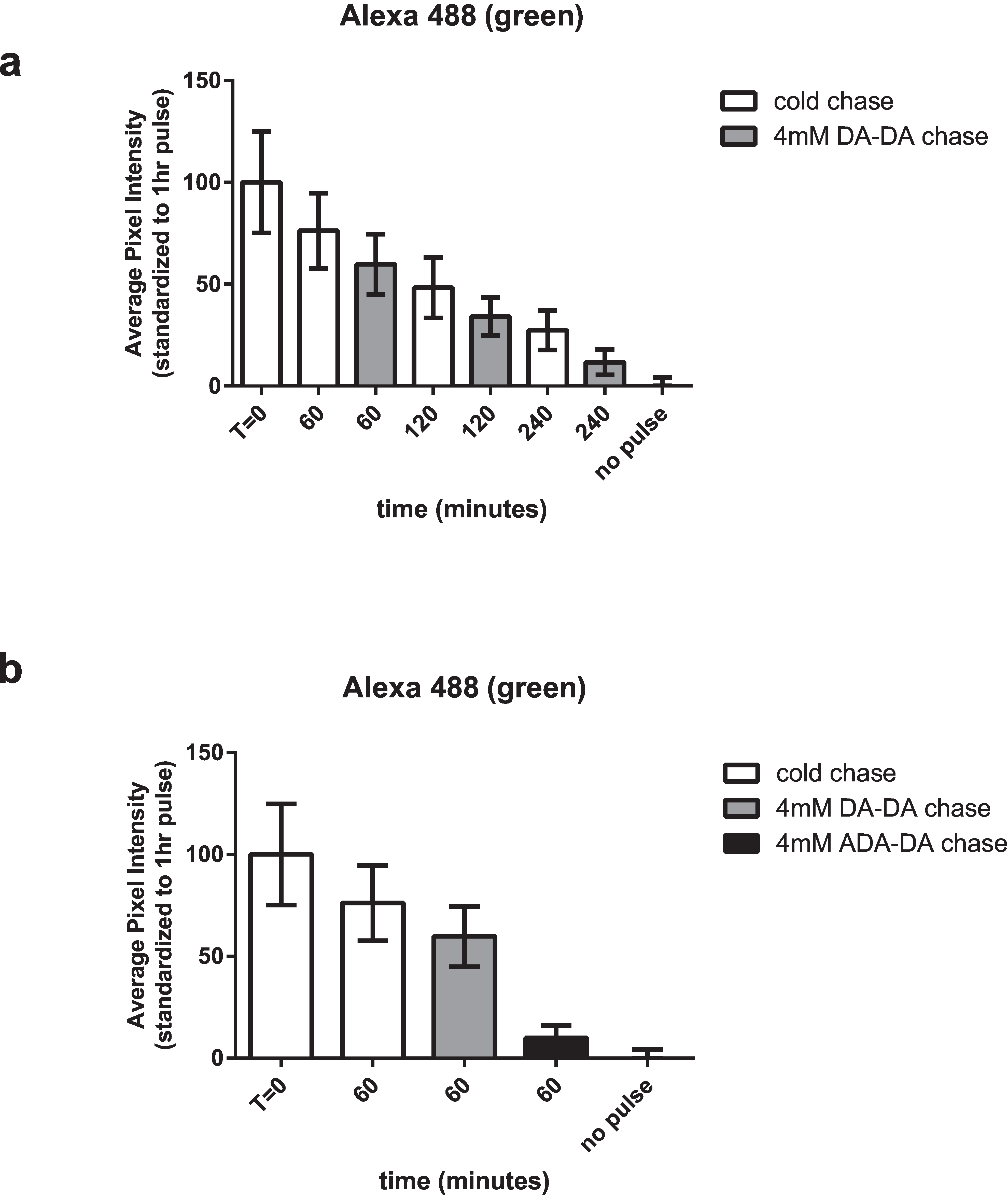

Supplement: S11 Fig — (a) Pulse chase experiments in Chlamydia with competitive, native dipeptide (DA—DA) supplemented into fresh medium and added to clickable, EDA—DA labeled inclusions. The inclusions that are sequentially pulsed with EDA—DA and DA-DA in order to show the effect that lengthy exposures to native DA-DA pools has on the loss of EDA—DA signal over time. (b) The same pulse chase experiment presented in panel a, but carried out over one hour and comparing the effects of exogenously added ADA-DA on loss of EDA-DA fluorescence. The addition of ADA—DA results in a drastic decrease in EDA—DA signal, most likely because incorporated azide containing ADA—DA outcompetes the Alexa Fluor 488 azide during the click chemistry reaction, i.e. most of the EDA—DA labeled PG is captured by ADA—DA labeled PG instead of the Alexa Fluor 488 that is used for the read-out of the EDA—DA labeled PG. For both experiments, average fluorescence values were calculated for each treatment group at the indicated time points, subsequent to chases. Each bar represents the average fluorescent pixel intensities of ~150 chlamydial inclusions pooled from two independent experiments. Error bars represent the standard deviation of the mean for each sample group. Chlamydia PG and MOMP were labeled as described in Fig 2. (TIF) [file ppat.1005590.s011.tif]
